# Tenascin-C promotes bladder cancer progression and its action depends on syndecan-4 and involves NF-κB signaling activation

**DOI:** 10.1186/s12885-022-09285-x

**Published:** 2022-03-04

**Authors:** Zhenfeng Guan, Yi Sun, Liang Mu, Yazhuo Jiang, Jinhai Fan

**Affiliations:** 1 Department of Urology, Shaanxi Provincial People’s Hospital, Xi’an, 710068 China; 2 Department of Urology, The First Affiliated Hospital of Xi’an Jiaotong University, 277 Yanta West Road, Xi’an, 710061 People’s Republic of China; 3 Department of B ultrasound, Shaanxi Provincial People’s Hospital, Xi’an, 710068 China

**Keywords:** Bladder cancer, Tenascin-C, Syndecan-4, progression, NF-κB

## Abstract

**Background:**

Bladder Cancer (BCa) is a severe genitourinary tract disease with an uncertain pathology. Increasing evidence indicates that the tumor microenvironment plays a decisive role with respect to cancer progression, and that this is driven by tumor cell interactions with stromal components. Tenascin-C (TN-C) is an important extracellular matrix (ECM) component, which has been reported to be involved in other types of cancer, such as breast cancer. The expression of TN-C in BCa tissue has been reported to be positively associated with the BCa pathological grade, yet the presence of urine TN-C is considered as an independent risk factor for BCa. However, the role of TN-C in BCa progression is still unknow. Thus, the object of the present investigation is to determine the role of TN-C in BCa progression and the involved mechanism.

**Methods:**

In this study, expression of TN-C in BCa tissue of Chinese local people was determined by IHC. Patients corresponding to tumor specimens were flowed up by telephone call to get their prognostic data and analyzed by using SPSS 19.0 statistic package. In vitro mechanistic investigation was demonstrated by QT-qPCR, Western Blot, Plasmid transfection to establishment of high/low TN-C-expression stable cell line, Boyden Chamber Assay, BrdU incorporation, Wound Healing, laser scanning confocal microscopy (LSCM) and ELISA.

**Results:**

TN-C expression in BCa tissue increases with tumor grade and is an independent risk factor for BCa patient. The in vitro investigation suggested that TN-C enhances BCa cell migration, invasion, proliferation and contributes to the elevated expression of EMT-related markers by activating NF-κB signaling, the mechanism of which involving in syndecan-4.

**Conclusions:**

Expression of TN-C in BCa tissues of Chinese local people is increased according to tumor grade and is an independent risk factor. TN-C mediates BCa cell malignant behavior via syndecan-4 and NF-κB signaling. Although the mechanisms through which syndecan-4 is associated with the activation of NF-κB signaling are unclear, the data presented herein provide a foundation for future investigations into the role of TN-C in BCa progression.

**Supplementary Information:**

The online version contains supplementary material available at 10.1186/s12885-022-09285-x.

## Background

Bladder cancer (BCa) accounts for 90-95% of urothelial carcinomas and is the most common urinary tract malignancy [1]. Almost 80% of BCa cases are initially diagnosed as non-muscle invasive BCa (NMIBC), which has a better prognosis; however, some of these tumors progress to muscle invasive BCa (MIBC). Even with surgical interventions, 30% of BCa cases become invasive [2] and are thus associated with a worse patient prognosis [3]. The remaining 20% of BCa cases are MIBC at initial diagnosis and have a less favorable prognosis, as 5% of patients have metastatic BCa [2]. The complete resection of all tumor tissue by transurethral bladder tumor resection (TURBT) is recommended for NMIBC, followed by chemotherapeutic instillation [4]. However, for specific types of BCa, such as T1G3 or carcinoma in situ (CIS), specific treatments are available. Radical cystectomy (RC) with extended lymphadenectomy is considered the standard treatment for MIBC [2], followed by cisplatin-based adjuvant chemotherapy. Two different pathological pathways [5–7] are considered to contribute to MIBC and this is responsible for the different prognoses between initially diagnosed MIBC and MIBC that is derived from NMBIC [8]. Therefore, improving our understanding of the mechanisms through which BCa progression occurs is warranted in order to establish more effective therapies for BCa.

The tumor microenvironment differs from normal tissue [9–11] and contributes to cancer progression. There is an interplay between cancer cells and stromal components, such as fibroblasts, macrophages and fibronectin, and the initiation of fibrosis is considered to be associated with tumor recurrence, drug-resistance and poor prognosis [10, 12–14]. As a vital component of the extracellular matrix (ECM) in the tumor mass, tenascin-C (TN-C) may have multifaceted and complicated roles in tumor progression.

TN-C is large (~ 300 kDa) as an intact monomer and ~ 1800 kDa when assembled as a hexamer [15]. Following initial identification in gliomas in 1980 s[16], TN-C has since been found to be expressed in head and neck squamous cell carcinoma, breast [17], prostate [18], thyroid [19], pancreatic [20] cancers, melanoma [21], gastric cancer [22] and osteosarcoma [23]. In the majority of these cancers, TN-C is considered to act as a tumor promoter and is associated with a worse prognosis. TN-C is considered to operate in the surrounding tumor microenvironment by binding to its receptor, annexin II [24, 25] or its co-receptor, syndecan-4/α5β 1[26–29], causing the loss of focal adhesions and mitogenesis, and increasing cell migration [30, 31]. However, the mechanisms underlying its binding to other receptors and initiating subsequent functions have not yet been established [32–34].

Four syndecan family members are found in mammals; among these, three (syndecan-1, 2 and 3) have a restricted tissue distribution. Syndecan-4 is expressed ubiquitously and is a member of the membrane-intercalated proteoglycans [35, 36]. Binding to fibronectin within two independent sites with syndecan-4 and α5β1 is key to the homeostasis of normal tissue [37], and involves the activation of downstream signals related to cytoskeletal organization and cell proliferation. TN-C has been reported to compete with the binding site of fibronectin with syndecan-4, and this interaction with syndecan-4 partially abrogates the effects of this co-receptor, as well as attenuates the interaction of syndecan-4 with fibronectin, enhancing tumor cell malignancy. This process also includes FAK and Rho signaling [37].

The activation of NF-κB signaling, manifested by the nuclear translocation of p65 [38–40], has been demonstrated with immunohistochemical (IHC) staining in BCa tissues and this has been reported to be positively associated with tumor progression. In addition, epithelial-mesenchymal transition (EMT) is another aspect of this signaling [41, 42], promoting BCa malignancy. Previous studies have suggested that TN-C is crucial for cancer progression [15] and urinary TN-C may be a useful biomarker of BCa progression [43–45].

The present study aimed to investigate the role of TN-C in BCa and elucidate the underlying mechanisms. In the present study, IHC analysis revealed that the expression of TN-C was significantly increased according to tumor grade. Further in vitro mechanistic analyses revealed that TN-C, as an ECM component, activated NF-κB signaling by binding with syndecan-4 to promote tumor progression. Although the mechanisms through which syndecan-4 activates NF-κB signaling remain unknown, the present study preliminarily clarified the mechanisms of TN-C in the process of BCa progression, as well as the potential signaling pathways involved. This provides an avenue for further research and may aid in the development of targeted drug design.

## Methods

### Tissue preparation and patient follow-up

BCa tissue samples (*n* = 57) were obtained from the Department of Urology, the First Affiliated Hospital of Xi’an Jiaotong University from Feb, 2010 to Aug, 2016 (32 males; age range, 39-78 years; mean age, 63.7 ± 7.5 years, Table 1). Pathological grading was performed by three independent hospital pathologists, and 15, 18 and 24 samples of grade I, II and III were noted, respectively, all of which were transitional cell carcinomas. Samples were fixed in 4% formalin and embedded in paraffin.

**Table 1:** Patient Cohort Characteristics of the Present Study

**Age**	
Range: 39-78 y; average age: 63.7 ± 7.5y	
**Patient No. in both Sex**	
M:32, F: 25;	
**Patient No. in different tumor Grade**	
G1:15; G2:18; G3:24;	
**Survival time**	
Range: 3-63.2 M, median:13 M, average: 20.03 M	
**Patient No. of Death during follow-up according to different TN-C expression**	
<average TN-C expression: 4; >average TN-C expression:18	

To assess TN-C expression and its association with tumor grade, TN-C and survival time were assessed, and the patients who provided the tumor samples were contacted by telephone. The survival data exhibited a normal distribution, as demonstrated by the Shapiro-Wilk test. The present study was approved by the Ethics Committee of Xi’an Jiaotong University and all patients involved provided signed informed consent.

### IHC staining of TN-C in BCa tissues

IHC staining was performed using a Dako Autostainer Plus system (Dako; Agilent Technologies, *Carpinteria, USA*). Tissues were pre-treated strictly according to the requirements of the manufacturer of the Dako Autostainer Plus system, including de-paraffinization and rehydration, and were subjected to 5-min pressure-cooking antigen retrieval, 15-min endogenous enzyme blocking, 60-min primary antibody (TN-C antibody, 1:300. cat: Sc-25,328; Santa Cruz Biotechnology, Inc., *Texas U.S.A*.) incubation at room temperature and a 30-min incubation at room temperature with Dako Cytomation EnVision-HRP reagent rabbit antibodies (1:200; Dako; Agilent Technologies, *Carpinteria, USA*). Signals were measured according to substrate hydrogen peroxide using DAB as a chromogen followed by hematoxylin counterstaining. Negative controls were prepared by omitting the primary antibody. Stained (brown) cells were quantified by counting the positive cells × 100/total cells in 10 random microscopic (Olympus, Japan) (× 400) fields in each section.

### Cells and cell culture

The human BCa cell lines, 5637, T24, RT4, J82 and UM-UC-3, were obtained from ATCC (Manassas, *VA, USA*) with 253 J as an exception, which was a gift from Professor Jer-Tsong Hsieh. RPMI-1640 (cat. no.31870082 for 5637 cells) and DMEM (cat. no.11054001 for the other cell lines) were obtained from Invitrogen Thermo Fisher Scientific, Inc. (*Carlsbad, USA*), and the medium was supplemented with 10% FBS (Invitrogen; Thermo Fisher Scientific, *California, USA*). Cells were cultured in 5% CO2 at 37 °C. Incubators used were from Thermo Fisher Scientific, Inc. (Carlsbad, USA).

### Reverse transcription-quantitative PCR (RT-qPCR)

Total RNA was isolated from the frozen tissues and cell lines using TRIzol® reagent (Invitrogen; Thermo Fisher Scientific, Invitrogen, *California, USA*) and quantified by reading the absorbance at 260 nm. RNA (2 μg) was reverse-transcribed using the Revert Aid™ First Strand cDNA Synthesis kit (Invitrogen; Thermo Fisher Scientific, *California, USA*.) according to the manufacturer’s protocol.

For qPCR, the SYBR® Premix Ex Taq™ II system (Takara Biotechnology Co., Ltd.) and a Bio-Rad CFX96™ Real-time system (Bio-Rad Laboratories, Inc., *California, USA*) were used. Subsequently, 12.5 μl SYBR® Premix Ex Taq™ II, 1 μl primer (10 μM, primers; Table 2), 200 ng cDNA and 9.5 μl double de-ionized water were mixed. Pre-degeneration was then conducted at 95 °C, 30 s, for one repeat, and PCR was performed at 95 °C for 5 s followed by incubation at 60 °C for 30 s, and 35 repeats. Dissociation was then performed at 95 °C for 15 s followed by incubation at 60 °C for 30 s, and a further incubation at 95 °C for 15 s and data collection. GAPDH was used as a loading control.

**Table 2:** Information of the antibodies

Gene ID	Antibody	Dilutions	Species	Supplied by
NM_004360.3	E-Cadherin	1:600	homo	*Santa Cruz*
NM_001792.3	N-Cadherin	1:300	homo	*Santa Cruz*
NM_003380.3	Vimentin	1:300	homo	*Santa Cruz*
NM_005985.3	Snail1	1:400	homo	*Santa Cruz*
NM_001145138.1	P65	1:300	homo	*Santa Cruz*
NM_004530.4	MMP2	1:400	homo	*Santa Cruz*
NM_004994.2	MMP9	1:400	homo	*Santa Cruz*
NM_002046.4	GAPDH	1:15000	homo	*Abcam*
NM_002999.3	Syndecan-4	1:400	homo	*Santa Cruz*
NM_002160.3	Tenascin-C (For WB)	1:300	homo	*Santa Cruz*
	Tenascin-C (peptide)		homo	*Millipore*
	Tenascin-C (For functional blocking)		homo	*NOVUS*

### Western blot analysis

The cells are harvested when 80% confluent and washed with cold PBS three times. Total cellular protein lysates were prepared with RIPA buffer [50 mM Tris (pH 8.0), 150 mM NaCl, 0.1% SDS, 1% NP40 and 0.5% sodium deoxycholate] containing proteinase inhibitors (1% cocktail and 1 mM PMSF, Sigma-Aldrich; Merck KGaA, *St Louis, MO*). Protein (30 μg) was separated with 6% (for TN-C) or 10% (for others) SDS-PAGE and transferred to nitrocellulose membranes. The membranes were blocked at room temperature for 1 h with 5% skimmed milk in Tris-buffered saline without Tween-20 (pH 7.6, TBS). Polyclonal antibody against TN-C was applied (1:300 dilution; Table 2) with 5% skimmed milk in TBS at 4 °C overnight, followed by washing with TBST (with Tween-20, pH 7.6). The membranes were then incubated with secondary antibodies (Licor, Rockford, *IL, USA*) coupled to the primary antibody at room temperature in the dark for 1 h. The membranes were then washed in a dark room, dried with neutral absorbent paper, and scanned using an Odyssey detection system (Licor, Rockford, *IL, USA*). Nuclear protein was prepared using a kit (cat. no. BSP001; Sangon Biotech, Shanghai, PRC.) according to the manufacturer’s protocol. GAPDH (for total cell fractions) and Histone H1 (for nuclear fractions) were used as loading controls.

### Boyden chamber and wound healing assay

Migration and invasion were examined using a Boyden chamber assay (Millipore, Sigma, *Massachusetts, USA*). For the migration assay, 0.2 ml FBS-free DMEM suspension with 1 × 10^4^ cells was added to the upper chamber and 0.8 ml FBS-free DMEM was then added to the lower chamber. Following 24 h of incubation (5% CO2, 37 °C), the chambers were washed with PBS (pH 7.4) three times to remove the attached cells in the upper chamber. Prior to staining (25 min, room temperature) with crystal violet (0.01% in ethanol), the cells were fixed with 4% formalin for 15 min (room temperature) and washed three times. The crystal violet-stained cells were under an inverted microscope and five visuals fields were randomly taken acquired under a × 200 objective, and cell counts were averaged. For the invasion assay, suspension in the upper chamber contained 0.2 ml FBS-free DMEM/Matrigel = 8/1 (Matrigel; Sigma-Aldrich; Merck KGaA Sigma, *St Louis, MO*) and 1 × 10^4^ cells were incubated (5% CO2, 37 °C) for 36 h. The cells were treated as described for the migration assay.

Wound healing was assessed by scratching 6-well dishes with a 10-μl pipette tip when the cells were 80% confluent. Scratch widths were compared at 0, 12 and 36 h.

### Preparation of stable clone cell lines

PsiCHECK-2^TN-C^ plasmids (plasmid 26,995, http://www.addgene.org) and a vector were transfected into the 5637 and 253 J (TN-C negative) BCa cell lines, respectively. Lipofectamine® 2000 (Invitrogen; Thermo Fisher Scientific, *California, USA*) was used for transfection according to the manufacturer’s instructions and stable cell clones highly expressing TN-C were selected by quantification with Western blot and RT-qPCR analyses.

Short hairpin RNA (shRNA/Sc) to knock down TN-C expression in the T24 and J82 cells (TN-C positive) was used. pGPU-6-GFP^TN-C/Sc^ was transfected into the cells as mentioned above. Low-TN-C expressing lines were selected using G418 (600 μg/ml), and western blot analysis and RT-qPCR was used to assess the shRNA effects. siRNA to knock down syndecan-4 expression was also used (Table 3). The protocol for transfection using Lipofectamine® 2000 was the same as that mentioned above.

**Table 3:** Primers for Real-time PCR and siRNA/shRNA

Gene ID	Gene	Primers	
NM_001145138.1	*P65*	F	ACG AAT GAC AGA GGC GTG TAT AAG G
		R	CAG AGC TGC TTG GCG GAT TAG
NM_002046.4	*GAPDH*	F	AAC AGC GAC ACC CAT CCT C
		R	CAT ACC AGG AAA TGA GCT TGA CAA
NM_004360.3	*E-Cadherin*	F	TGC CCA GAA AAT GAA AAA GG
		R	GTG TAT GTG GCA ATG CGT TC
NM_001792.3	*N-Cadherin*	F	ACA GTG GCC ACC TAC AAA GG
		R	CCG AGA TGG GGT TGA TAA TG
NM_003380.3	*Vimentin*	F	GAG AAC TTT GCC GTT GAA GC
		R	GCT TCC TGT AGG TGG CAA TC
NM_005985.3	*Snail1*	F	ACC CCA ATC GGA AGC CTA ACT
		R	GGT CGT AGG GCT GCT GGA A
NM_004530.4	*MMP2*	F	CTC ATC GCA GAT GCC TGG AA
		R	TTC AGG TAA TAG GCA CCC TTG AAG A
NM_004994.2	*MMP9*	F	TGA CAG CGA CAA GAA GTG
		R	CAG TGA AGC GGT ACA TAG G
NM_002999.3	*Syndecan-4*	F	CCA GTT TGA TGT TGC TGG GTG GTT
		R	AGC CCT AGA GCC TGA AGA AAG CAA
	***Syndecan-4 siRNA***	Si	5 -AAG GCC GAT ACT TCT CCG GAG-3
		Sc	5 -AAG GCT CTC CGG AGC GATA CT-3
NM_002160.3	*Tenascin-C*	F	AGC TTC CAA GAA ACA CCA CTT C
		R	CCA TCC CAG CCA ACC TCA
	***Tenascin-C shRNA***	F	5′-CAC CGC ACC TGA AGG CCT GAA ATT CTT CAA GAG AGA ATT TCA GGC CTT CAG GTG CTT TTT TG-3’
		R	5′-GAT CCA AAA AAG CAC CTG AAG GCC TGA AAT TCT CTC TTG AAG AAT TTC AGG CCT TCA GGT GC-3’

### BrdU incorporation assay

BrdU was added to the cell medium (3 μg/ml) after the cells reached 60-70% confluency on coverslips, followed by incubation (room temperature) for 4 h. The coverslips were then rinsed three times with PBS for 10 min to remove free BrdU and the samples were fixed in 4% paraformaldehyde for 45 min, followed by rinsing five times with PBS for 20 min. Subsequently, 0.1% Triton X-100 was added to destroy the cell membrane (15 min) and 2 M HCl (25 min) were added to separate DNA into single strands for primary antibody access to incorporated BrdU. Before blocking non-specific epitopes with 10% BSA for 20 min, the cells were rinsed three times with PBS for 10 min to remove HCl and Triton. Subsequently, 10% BSA with anti-BrdU antibody (1:200, cat 8152 Waltham, *MA USA*) was added followed by incubation overnight at 4 °C. The following day, the cells were rinsed five times with PBS to remove free antibody, and were incubated with TRITC-labeled secondary antibody for 1 h at room temperature and rinsed a further three times with PBS to remove free antibody. The fluorescence intensity of TRITC was monitored using a Super Micro Orifice Plate Spectrophotometer (BioTek Instruments, *Vermont, USA*) at 547 nm.

### ELISA

TN-C in the cell medium was measured by ELISA. Briefly, cell (different groups) media were collected and examined by ELISA according to the kit instructions (Human TN-C ELISA kit, Shanghai Westang Biological Technology Co., Ltd. *Shanghai, PRC*), and optical density was measured at 450 nm. Data are expressed in μg/ml.

### Immunofluorescence staining for the nuclear translocation of NF-κB

The prepared cells were washed three times in cold PBS (pH 7.4) and fixed with 4% paraformaldehyde for 15 min, followed by permeabilization in 0.5% Triton X-100 for 10 min and blocking with 1% BSA for 1 h. Rabbit anti-human p65 in 1% BSA was added to the medium and incubated overnight at 4 °C. Mouse anti-rabbit TRITC (red) IgG antibody (Santa Cruz Biotechnology, *Texas, USA*) diluted 1:100 in blocking buffer was added to the medium and incubated (room temperature) for 1 h. The cells were then washed with cold PBS three times and cell nuclei were stained with DAPI (10 μg/ml, Sigma-Aldrich; Merck KGaA Sigma, *St Louis, USA*) for 3 min. Cells were observed under an Image Pro Plus System mounted on a fluorescence microscope (Olympus Corporation, *Tokyo, Japan*).

### Other reagent and experiments

TN-C peptide (Millipore Sigma, *Massachusetts, USA*) was an exogenous TN-C added to the media (1 μg/ml), and TN-C-neutralizing antibody (1 μg/ml) (3358-TC, Novus Biologicals, *Briarwood Avenue, USA*;) was used to neutralize TN-C in the media.

Statistical analysis Statistical analysis was performed using the SPSS 19.0 statistic package (SPSS, Inc., *Chicago, USA*.). For comparisons among different groups, one-way ANOVA was used, and for comparisons between two different groups, the student’s t-test was used. A Shapiro-Wilk test was used to confirm the distribution of survival time of the BCa patients. *P* < 0.05 was considered to indicate statistically significant differences.

## Results

### TN-C expression in BCa tissue increases with tumor grade and is an independent risk factor for BCa

**Fig. 1:**
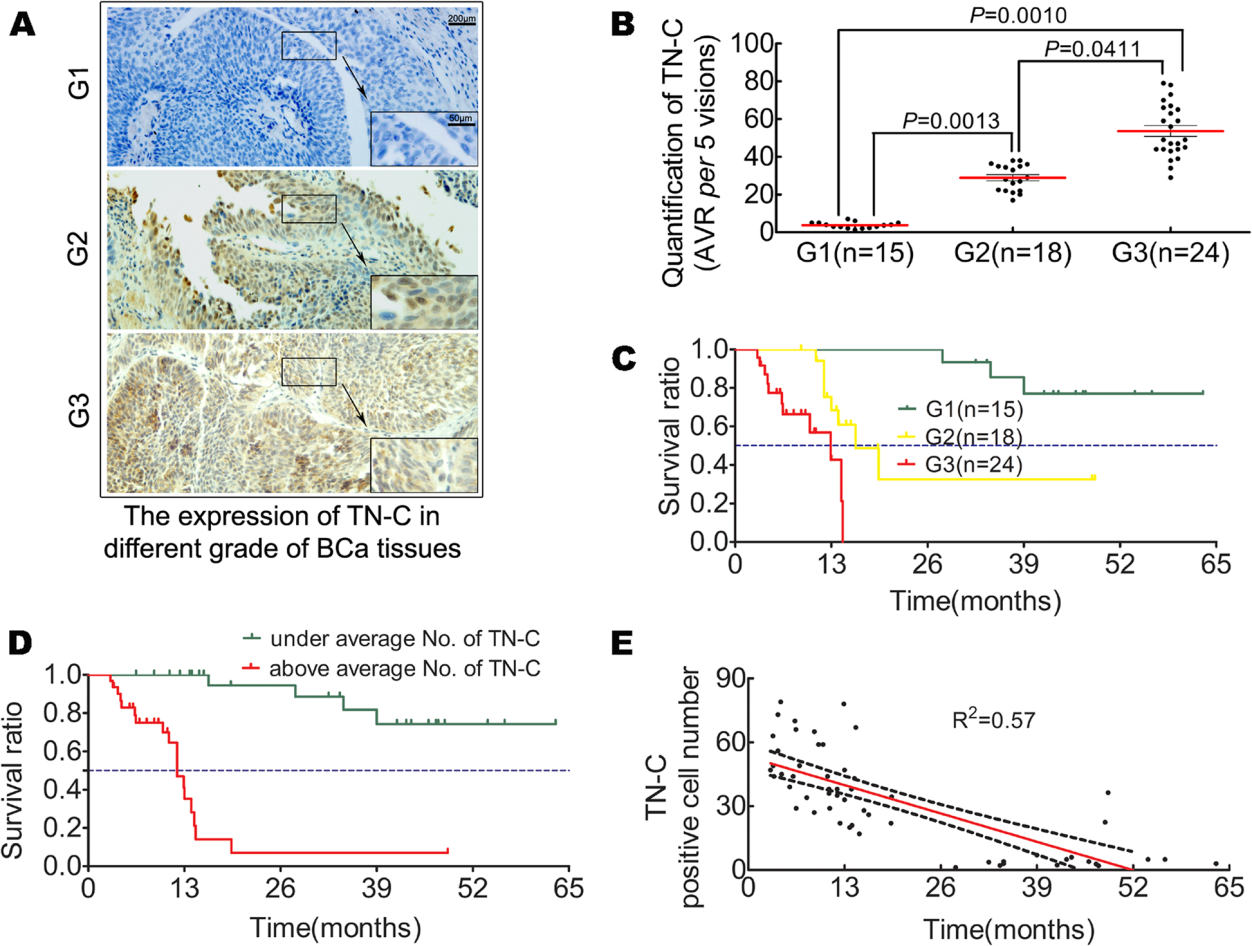
TN-C expression in BCa tissue and clinical significance. **A** Representative figures of IHC staining for TN-C in different BCa tissues; **B** Quantification of IHC staining for TN-C in different BCa tissues, indicating elevated expression of TN-C according to BCa tumor grade, *P* < 0.05; **C** Kaplan-Meier analysis suggests that tumor grade is an independent risk factor for BCa; **D** Kaplan-Meier analysis indicates that TN-C expression in BCa tissue is an independent risk factor for BCa, based on normal survival distribution demonstrated by the Shapiro-Wilk test. Patients are stratified by average TN-C positive cells: green line indicates under average; red line indicates above average, *P* < 0.05; **E** Correlation analysis suggests that tumor-free survival is negatively associated with TN-C expression in BCa patients, *P* < 0.05

To examine the expression of TN-C in BCa tissues in local Chinese patients and its significance in clinical practice, TN-C expression was measured in BCa tissues obtained from Chinese patients. As shown in Fig. 1A and B, TN-C expression across different grades of BCa tissues differed and increased with the tumor grade. In addition, it was observed that the BCa tumor grade was an independent risk factor for BCa patients (Fig. 1C). To examine TN-C expression and patient survival, the patients were stratified into the high or low TN-C expression groups. As shown in Fig. 1D, TN-C expression exceeded the mean, suggesting a poor survival, and these data are in agreement with previous findings [46]. Finally, an association analysis suggested that TN-C expression in BCa tissue was negatively associated with tumor-free survival (Fig. 1E).

### Preparation of stable high and low TN-C-expressing cell lines

**Fig. 2:**
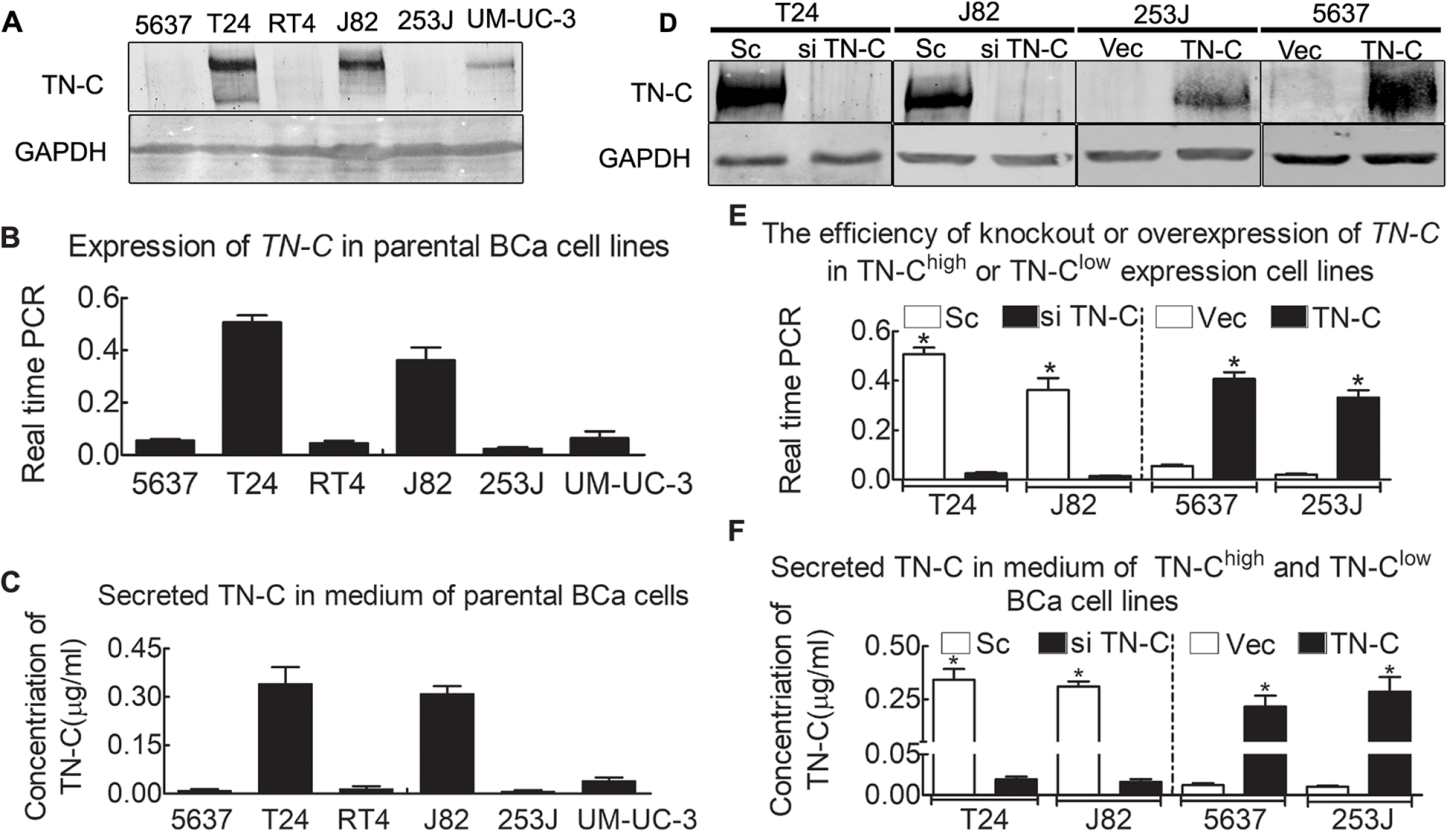
TN-C expression in BCa cell lines. **A** Western blot of TN-C expression in parental BCa cell lines; GAPDH was a loading control, indicating different expression profile, with the strongest expression for T24 and J82, accompanied by the weakest for 253 J and 5637; **B** Real time PCR indicates TN-C expression in parental BCa cell lines, *P* < 0.05, suggesting expression differences; **C** ELISA depicting TN-C in parental BCa cell line medium, indicating TN-C also as a component of extracellular matrix (*P* < 0.05). This expression pattern got consistent with protein and mRNA indicated by Western Blot and real time PCR; **D** Western blot indicates efficiency of TN-C knockdown (T24^si TN-C^*Vs* T24^sc^ and J82^si TN-C^ Vs J82^sc^) or TN-C over-expression (5637^TN-C^*Vs* 5637^Vec^ and 253J^TN-C^*Vs* 253J^Vec^), GAPDH was a loading control; **E** Real time PCR indicates the efficiency of TN-C knockout (T24^si TN-C^*Vs* T24^sc^ and J82^si TN-C^ Vs J82^sc^) or TN-C over-expression (5637^TN-C^*Vs* 5637^Vec^ and 253J^TN-C^*Vs* 253J^Vec^), *P < 0.05; **F** ELISA data depicting TN-C in medium from cells with TN-C knocked out or with TN-C stably overexpressed, *P < 0.05

As it was suggested that TN-C expression may drive BCa progression, TN-C expression was measured in BCa cell lines of different tumor grades. It is known that TN-C is secreted into the ECM. Thus, the present study measured TN-C expression in cell media to monitor this secretion. The data indicated the diverse expression of TN-C in BCa cell lines (Fig. 2A and B). The T24 and J82 cells exhibited a higher TN-C expression, while the 5637 and 253 J cells exhibited a lower TN-C expression. The results of ELISA were in agreement with those of western blot analysis and RT-qPCR, suggesting that TN-C may function as a secreted protein (Fig. 2C).

To elucidate the mechanisms through which TN-C contributes to BCa progression, TN-C expression was silenced in BCa cell lines, and malignancy and proliferation were monitored. Briefly, the T24 and J82 cells, TN-C-positive cell lines, were used and TN-C was knocked down with shRNA/Sc. The 5637 and 253 J cells, TN-C-negative cell lines, were then manipulated to overexpress TN-C with a plasmid as described in *Materials and methods*. The TN-C-knockdown or overexpression (Fig. 2D-F) indicated successful transfection efficiencies.

### TN-C enhances the migration, invasion and proliferation of BCa cell lines

**Fig. 3:**
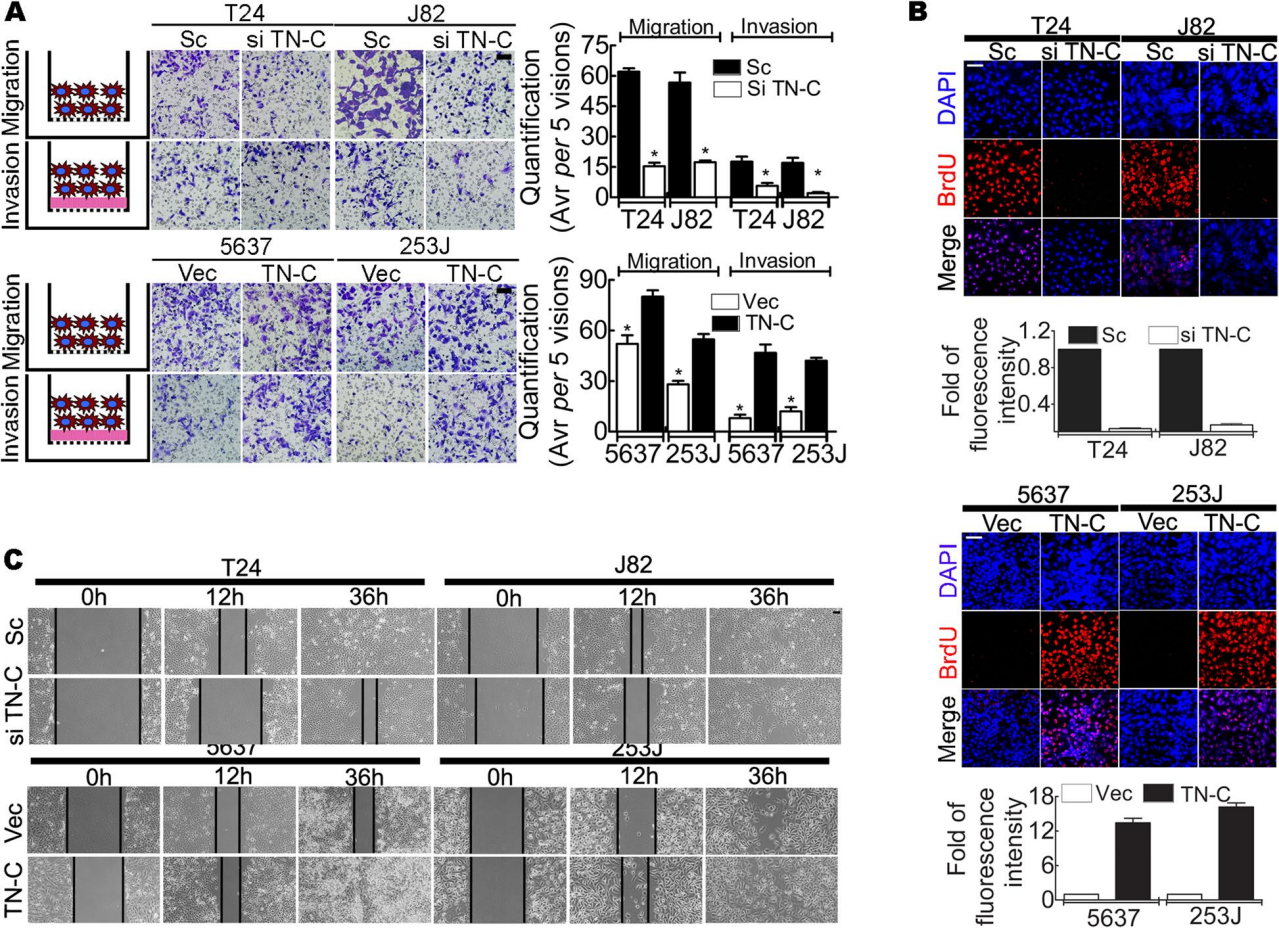
TN-C contributes to BCa cell line proliferation, migration, and invasion. **A** Boyden chamber assay, indicating that TN-C contributes to tumor cell migration and invasion. Left: cartoon of tumor cell with or without Matrigel (pink area), middle: representative figures, Bar: 100 μm; right: quantification, *P < 0.05; **B** BrdU incorporation, indicating that TN-C contributes to tumor cell proliferation. Upper: representative figures, bar: 100 μm; lower: relative fluorescent intensity; **C** Wound healing analysis at 0, 12, and 36 h suggesting that TN-C leads reduces wound healing time, Bar: 100 μm

TN-C contributes to tumor migration, invasion and proliferation in diverse tumors, including breast cancer, melanoma and pancreatic cancer. The data of the present study demonstrated that TN-C was vital for BCa progression. TN-C was knocked down in the T24 and J82 cell lines, and a reduced cancer cell migration and invasion (Fig. 3A, upper panels), an attenuated proliferation (Fig. 3B, upper panels), and a delayed and prolonged wound healing (Fig. 3C, upper panels) were noted. TN-C overexpression in 5637 and 253 J cells enhanced migration and invasion (Fig. 3A, bottom panels), increased proliferation (Fig. 3B, bottom panels) and accelerated wound healing (Fig. 3C, bottom panels).

Research suggests a complex role for TN-C as an ECM component [15]; however, in the present study, the TN-C staining data from BCa tissues indicated that the ECM deposition of TN-C occurred beyond the cytoplasm. Thus, tumor cells exposed to exogenous TN-C may be modified. Human TN-C peptide (which was used as exogenous TN-C) was added to the media of TN-C-negative cell lines (T24^siTN-C^, J82^siTN-C^, 5637^Vec^ and 253J^Vec^), and human TN-C-neutralizing antibody was added to TN-C-positive cell lines (T24^Sc^, J82^Sc^, 5637^TN-C^ and 253J^TN-C^). Boyden chamber, wound healing and BrdU incorporation assays were then performed to assessed malignancy.

**Fig. 4:**
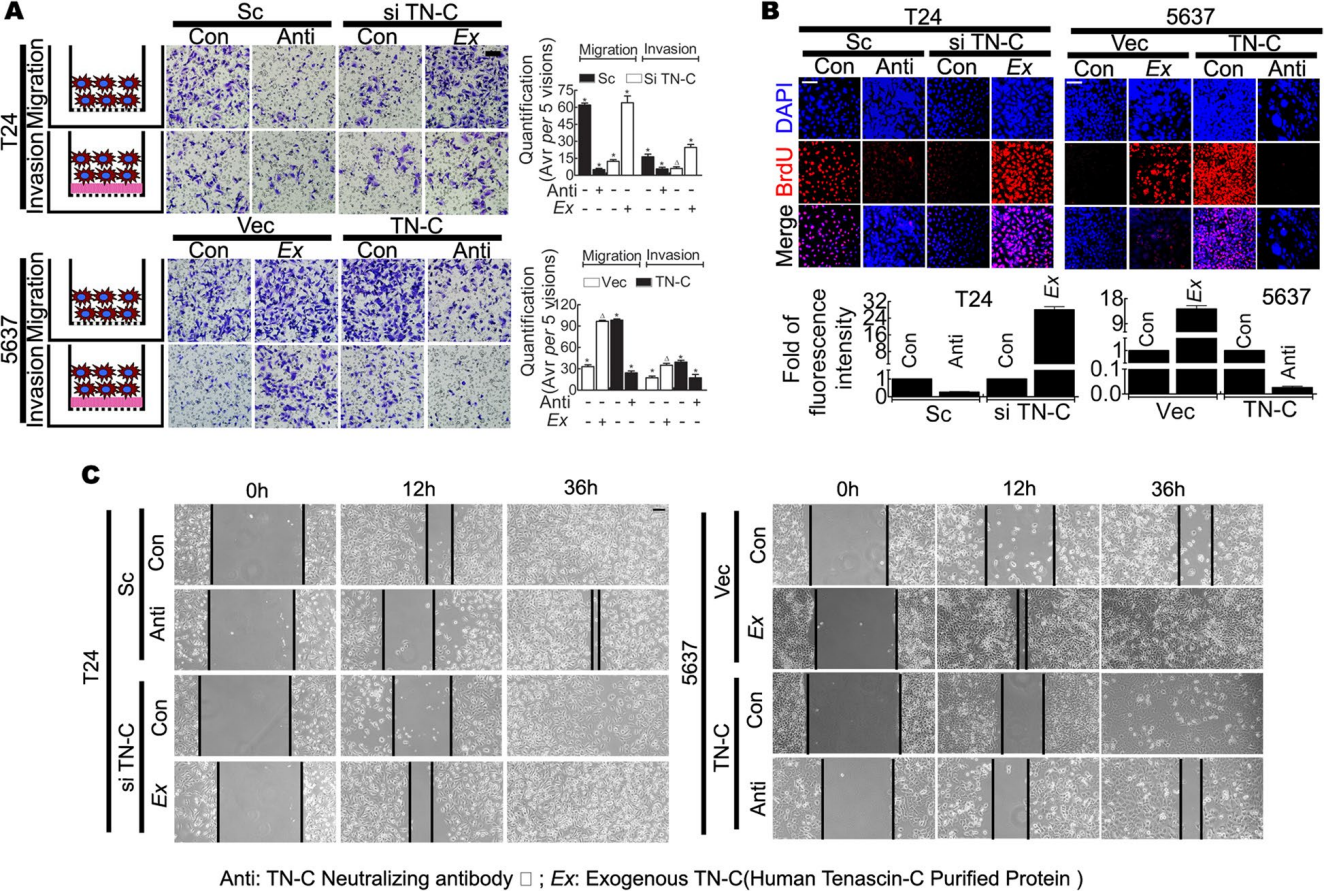
TN-C promotes tumor invasion, migration and proliferation as a secreted glycoprotein. **A** Boyden chamber assay indicates that TN-C promotes BCa cell migration and invasion as secreted glycoprotein. Left: cartoon of tumor cell with or without Matrigel (pink area); Middle: representative figures, Bar: 100 μm; Right: quantification, *P* < 0.05. The exogenous TN-C (*Ex*) promotes tumor cell migration and invasion (T24^si TN-C^-*Ex* Vs T24^si TN-C^-con, 5637^Vec^-*Ex* Vs 5637^Vec^-con), whereas the neutralizing antibody of TN-C (Anti) attenuates this phenomenon (T24^sc^-Anti Vs T24^sc^-con, 5637^TN-C^-Anti Vs 5637^TN-C^-con); ^*^P < 0.05, ^△^*P* > 0.05; **B** BrdU incorporation suggests that TN-C promotes BCa cell proliferation as secreted glycoprotein. Upper: representative figures, Bar: 100 μm; Lower: relative fluorescent intensity. The exogenous TN-C (*Ex*) leads to tumor cell proliferation (T24^si TN-C^-*Ex* Vs T24^si TN-C^-con, 5637^Vec^-*Ex* Vs 5637^Vec^-con), and TN-C neutralizing antibody inhibits this (T24^sc^-Anti Vs T24^sc^-con, 5637^TN-C^-Anti Vs 5637^TN-C^-con); **C** Wound healing analysis indicates that TN-C promotes BCa cell wound healing also as secreted glycoprotein. *Ex* Vs con (T24^si TN-C^-*Ex* Vs T24^si TN-C^-con, 5637^Vec^-*Ex* Vs 5637^Vec^-con) indicates that exogenous TN-C reduces wound-healing time, and Anti Vs con (T24^sc^-Anti Vs T24^sc^-con, 5637^TN-C^-Anti Vs 5637^TN-C^-con) indicates that neutralizing antibody prolongs this duration. Bar: 100 μm

Exogenous TN-C (*Ex TN-C*, *Ex*) enhanced the migration, invasion and proliferation of 5637^Vec^ cells (Fig. 4 A-C: 5637^Vec^, Con vs. Ex), while the knockdown of TN-C attenuated T24 cell migration, invasion and proliferation. This effect was attenuated by exogenous TN-C (Fig. 4A-C: T24^siTN-C^, Con vs. Ex). TN-C-neutralizing antibody added to the media decreased the migration, invasion and proliferation of T24^Sc^ (Fig. 4A-C: T24^Sc^, Con vs. Anti) and 5637^TN-C^ (Fig. 4A-C: 5637^TN-C^, Con vs. Anti) cells.

### TN-C contributes to the elevated expression of EMT-related markers and the expression of MMP2/MMP9 by activating NF-κB signaling

**Fig. 5:**
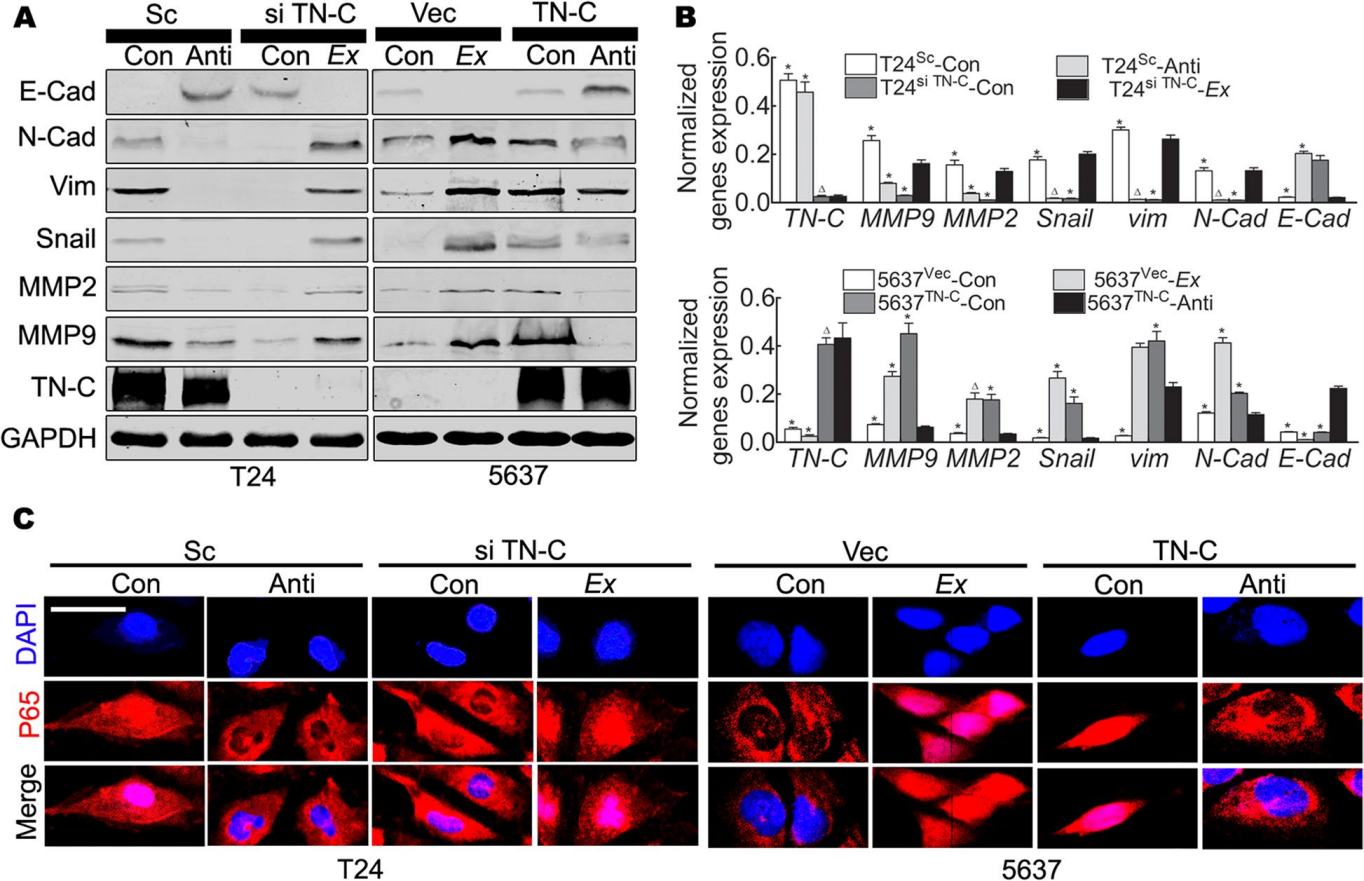
Secreted TN-C induces activation of NF-κB signaling, leading to EMT. **A** Western blot indicates that secreted TN-C induces upregulation of N-Cad, Vim, Snail, MMP_2_ and MMP_9_ and downregulation of E-Cad; GAPDH was a loading control; **B** Real time PCR agrees with Western blot, *P < 0.05, ^△^*P* > 0.05; **C** Immunofluorescent staining suggests that secreted TN-C contributes to nuclear translocation of NF-κB (P65), activating signaling, Bar: 20 μm

The data demonstrated that TN-C promoted BCa cell migration, invasion and proliferation; however, the underlying mechanisms were not clear. TN-C may perform this function as a component of the ECM, at least partially. In transitional cell carcinoma, the enhanced migration and invasion of tumor cells is often accompanied by EMT [47], and this can be used to monitor the malignant behavior of BCa cells. The activation of NF-κB has been causally linked to an invasive phenotype, and can directly or indirectly induce the expression of Snail, Slug, Twist, Zeb1 and Zeb2 [48], all of which are markers of EMT. Thus, it was hypothesized that the TN-C-induced effects may involve NF-κB signaling. The data from western blot analysis and RT-qPCR to monitor the expression of EMT-related markers, and immunofluorescence staining to quantify NF-κB signaling confirmed the data from the Boyden chamber and wound healing assays. In addition, the knockdown of TN-C in T24 cells confirmed the decreased expression of MMP2/MMP9, vimentin, N-cadherin and Snail, and this was accompanied by the elevated expression of E-cadherin (Fig. 5A and B, T24^Sc^-Con vs. T24^siTN-C^-Con), indicating the reversal of EMT.

Moreover, this process was reversed in 5637 cells overexpressing TN-C, which caused the elevated expression of MMP2/MMP9, vimentin, N-cadherin and Snail, and the decreased expression of E-cadherin (Fig. 5A and B, 5637^Vec^-Con vs. 5637^TN-C^-Con). The effect of knocking down TN-C in T24 cells was reversed with Ex TN-C (Fig. 5A and B, T24^siTN-C^-Con vs. T24^siTN-C^-Ex), and TN-C-neutralizing antibody significantly inhibited the expression of these genes in T24^Sc^ cells (Fig. 5A and B, T24^Sc^-Con vs. T24^Sc^-Anti). Similar data were obtained with the 5637 cells; Ex TN-C (Fig. 5A and B, 5637^Vec^-Con vs. 5637^Vec^-Ex) promoted EMT, and TN-C-neutralizing antibody inhibited this effect (Fig. 5A and B, 5637^TN-C^-Con vs. 5637^TN-C^-Anti).

In addition, TN-C induced the activation of NF-κB signaling [34]. p65 is the functional subunit of the NF-κB dimer (p65/p50), and the nuclear translocation of this subunit is considered to activate this signaling. As shown in Fig. 5C, TN-C induced the nuclear translocation of p65 in T24^siTN-C^ vs. T24^Sc^ cells and in 5637^Vec^ vs. 5637^TN-C^ cells, as well as in the T24^siTN-C^-Con vs. T24^siTN-^C-Ex and 5637^Vec^-Con vs. 5637^Vec^-Ex. However, the nuclear translocation of p65 was suppressed by the functional inhibition of TN-C (Fig. 5C, T24^Sc^-Con vs. T24^Sc^-Anti and 5637^TN-C^-Con vs. 5637^TN-C^-Anti).

### TN-C induced activation of NF-κB signaling is dependent on syndecan-4

**Fig. 6:**
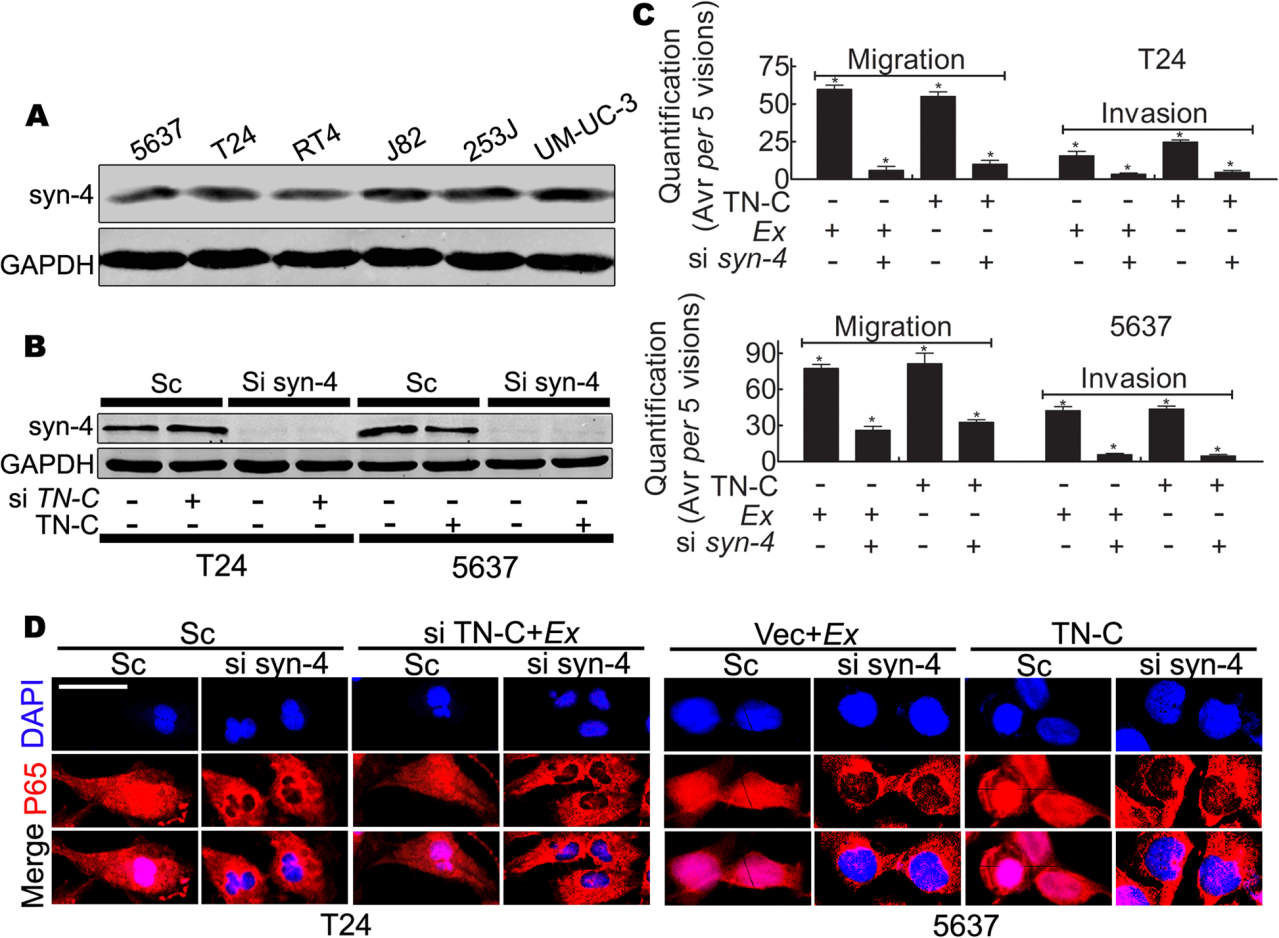
TN-C contributes to activation of NF-κB signaling depends on membrane receptor syndecan-4. **A** Western blot indicates the ubiquitous expression of syndecan-4 (syn-4) in parental BCa cell lines, GAPDH was a loading control; **B** Western blot indicates that there is no visible discrepancy expression of syn-4 in T24^sc^ Vs T24^siTN-C^ and 5637^Vec^ Vs 5637^TN-C^, suggesting that TN-C has no effect on the syn-4 expression in tumor cell line, and siRNA to knock down syn-4 significantly attenuated expression of syn-4 in both cell lines, indicating the perfect efficiency; **C** Quantification of Boyden chamber assay indicates knocking down syn-4 in both cell lines decreases the ability of migration and invasion in the presence of TN-C, ^*^P < 0.05; **D** Immunofluorescent staining indicates that TN-C induced nuclear translocation of NF-κB is diminished in the absent of syn-4, Bar: 20 μm

TN-C chiefly functions as a component of the ECM, indicating an interaction between TN-C and tumor cells [49]. Syndecan-4 has also been reported to be the receptor involved in these interactions. Briefly, syndecan-4 is considered as a co-receptor of syndecan-4/α5β1, which is crucial for cell adhesion. Interference with this co-receptor causes tumor cell proliferation and metastases; thus, the functions of TN-C may be dependent on this membrane receptor in the BCa cell line. To assess this, the present study measured the expression of syndecan-4 in all BCa cell lines. The results revealed that a significant syndecan-4 expression was observed in all lines (Fig. 6A). No apparent differences were observed among cell lines. The T24 and 5637 cells were selected to represent TN-C-positive and negative-cell lines, respectively, and the role of syndecan-4 was examined by knocking down its expression with siRNA.

Two TN-C related stable cell clones, T24^Sc/siTN-C^ and 5637^Vec/TN-C^ cells, were also subjected to syndecan-4 knockdown and the effects of TN-C on that expression were examined. The data indicated that syndecan-4 expression was knocked down in both cell lines; however, the knockdown of TN-C did not affect syndecan-4 expression (Fig. 6B).

To determine whether syndecan-4 knockdown in both cell lines can modify migration and invasion, a Boyden chamber assay was performed and syndecan-4 knockdown was noted to decrease the migration and invasion of both cell lines. The effects of the overexpression of TN-C (in 5637 cells) or the exogenous addition of TN-C (in T24^siTN-C^ and 5637^Vec^ cells) were attenuated (Fig. 6C).

In addition, syndecan-4 interference inhibited p65 nuclear translocation, blocking signal activation, as shown by immunofluorescence staining (Fig. 6D). The effects of the overexpression of TN-C (in 5637 cells) or the addition of Ex TN-C (in T24^siTN-C^ and 5637^Vec^ cells) were also blocked by syndecan-4 knockdown. Thus, TN-C promotes cancer cell line migration, invasion and proliferation, as well as the activation of NF-κB signaling, and these effects are dependent on syndecan-4.

## Discussion

The high recurrence rate of BCa is likely attributed to the interactions of tumor cells with the surrounding microenvironment to drive progression, metastasis and drug resistance. Macrophages from prostate cancer tissue can induce cancer phenotypes of normal prostate epithelial cells when co-cultured [50]. In addition, fibroblasts, inflammatory cells and the ECM play vital roles in cancer, among which, TN-C is the least extensively investigated.

TN-C has been reported to be crucial for embryogenesis, inflammation and wound healing, and functions in a similar manner in tumorigenesis. TN-C expression in cancerous tissues has been documented in various tumors and is considered to be an independent risk factor for patients with cancer. Consistent with the literature [51], TN-C expression data for BCa tissue in the present study suggested that it plays a positive role in BCa progression; however, TN-C expression across different BCa cell lines is diverse and is not associated with tumor grade. For example, in 5637 cells, TN-C expression is not observed in grade II BCa, and this may be explained by the different sources of TN-C. In BCa cell lines, cancer is the sole TN-C source, although tumor cell secretions or mesenchymal cells can also produce TN-C.

Previous studies have suggested a vital role of TN-C in tumor progression and the TN-C content in BCa cell lines is consistent with tumor cell TN-C expression (Fig. 2A and C). Modifying TN-C expression in BCa cell lines causes the same effect to the TN-C concentration in the corresponding BCa cell lines (Fig. 2D and F). Thus, secreted TN-C may be a primary source of TN-C tumor activity. TN-C-neutralizing antibody reduces TN-C overexpression, as does human TN-C peptide (Fig. 4). Thus, in BCa cell lines, TN-C executes its role mainly as a component of the ECM, perhaps by binding with membrane receptors. Investigations have confirmed that the co-receptor of syndecan-4/α5β1 is important for tumor cell adhesion to fibronectin of the ECM [28], and that interference with this function by TN-C decreases tumor cell adhesion and enhances metastasis and proliferation. Syndecan-4 is the sole syndecan family member that is ubiquitously expressed in the cell membrane. A number of downstream signals of syndecan-4 are known, including PKCα, PKCδ, PI3K/Akt and synectin [29, 52]. Previous studies have suggested that interference with the syndecan-4/α5β1 co-receptor in the cell membrane inhibits normal cell proliferation and enhances tumor cell proliferation; however, the mechanisms responsible for this remain unclear [26, 27].

The present study found that the knockdown of syndecan-4 expression attenuated TN-C-induced tumor migration, invasion and proliferation, suggesting that TN-C contributes to metastasis and proliferation in a manner that is dependent on syndecan-4. However, the mechanisms through which TN-C binds to membrane syndecan-4 and those underlying its association with metastasis and proliferation are not yet known. The enforced expression of TN-C or in the presence of Ex TN-C increased the levels of mesenchymal markers (elevated expression of vimentin, Snail, N-cadherin and MMP2/MMP9) and decreased the expression of E-cadherin (Fig. 5A and B) in the BCa cell lines, indicating that the alternative expression of these genes may be related to the activation of NF-κB signaling.

The present study provides evidence that in BCa cell lines, the activation of NF-κB signaling leads to EMT, manifested as previously depicted [53]. Immunofluorescence staining confirmed that the binding of TN-C, either by the enforced expression or exogenous TN-C, with syndecan-4 induced the nuclear translocation of p65, a process that was inhibited by syndecan-4 knockdown (Fig. 6D). Thus, the binding of TN-C with syndecan-4 induces the activation of NF-κB signaling, and promotes tumor cell metastasis and proliferation.

**Fig. 7:**
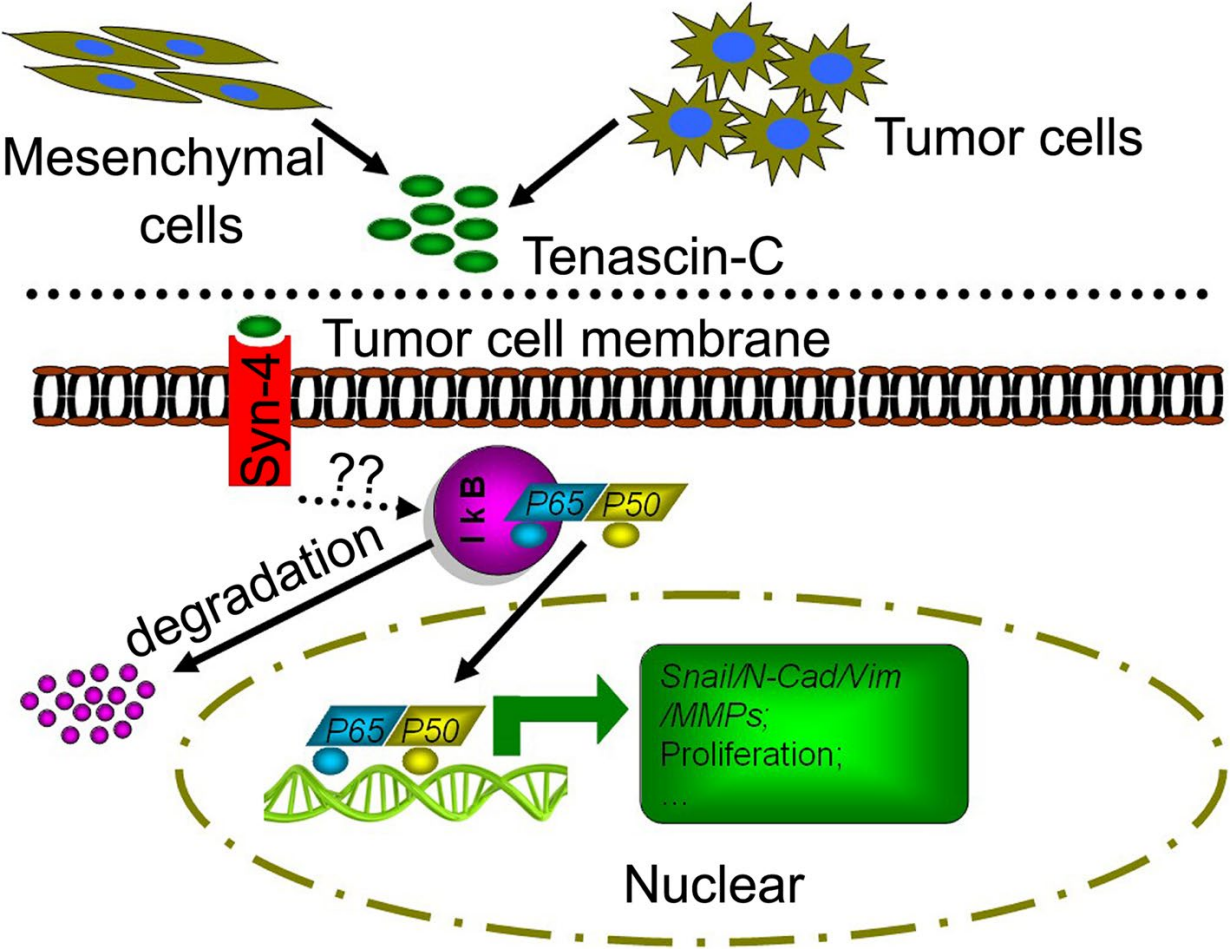
Summary of the present investigation. In the BCa microenvironment, TN-C may be secreted by diverse cells including tumor cells and other mesenchymal cells, e.g., fibroblast cells, etc. Thus, the secreted TN-C binds to the membrane receptor syndecan-4 and a critical downstream factor for this is NF-κB signaling. Binding of TN-C to syndecan-4 leads to activation of NF-κB signaling and subsequent enhanced ability of cells to migrate, invade, and proliferate as well as cause EMT. However, how syndecan-4 activates NF-κB signaling is not known

A summary of the present study is illustrated in Fig. 7. NF-κB is downstream of the PI3K/Akt pathways, suggesting that TN-C binding with syndecan-4 may induce the activation of NF-κB signaling is involved in PI3K/Akt pathway activation via the cytoplasmic domain of syndecan-4. Whether the binding of TN-C with syndecan-4 involves the co-receptor α5β1 remains unknown.

## Conclusion

In summary of the present study, our data suggested that TN-C promotes tumor cell metastasis and proliferation, and that this is dependent on syndecan-4 mediated activation of NF-κB signal activation, although the mechanism of which was still unknown. These data offer a solid foundation for future studies on the role of TN-C in BCa progression and suggest that TN-C may be a potential therapeutic target in the treatment of BCa.

## Supplementary Information

**Additional file 1.**

## Data Availability

All data generated or analyzed during this study are included in this published article (and its supplementary information files).

## Abbreviations

BCa: Bladder Cancer
CIS: Carcinoma in situ
EMT: Epithelial-mesenchymal transition
ECM: Extracellular Matrix
IHC: Immunohistochemical
MIBC: Muscle invasive bladder cancer
NMIBC: Non-muscle invasive bladder cancer
RC: Radical cystectomy
TN-C: Tenascin-C
TURBT: Transurethral bladder tumor resection
