# Supplementary figures and images for: Tenascin-C promotes bladder cancer progression and its action depends on syndecan-4 and involves NF-κB signaling activation
Source: BMC Cancer. 2022 Mar 4;22:240. doi: 10.1186/s12885-022-09285-x (PMC8896393; doi:10.1186/s12885-022-09285-x)

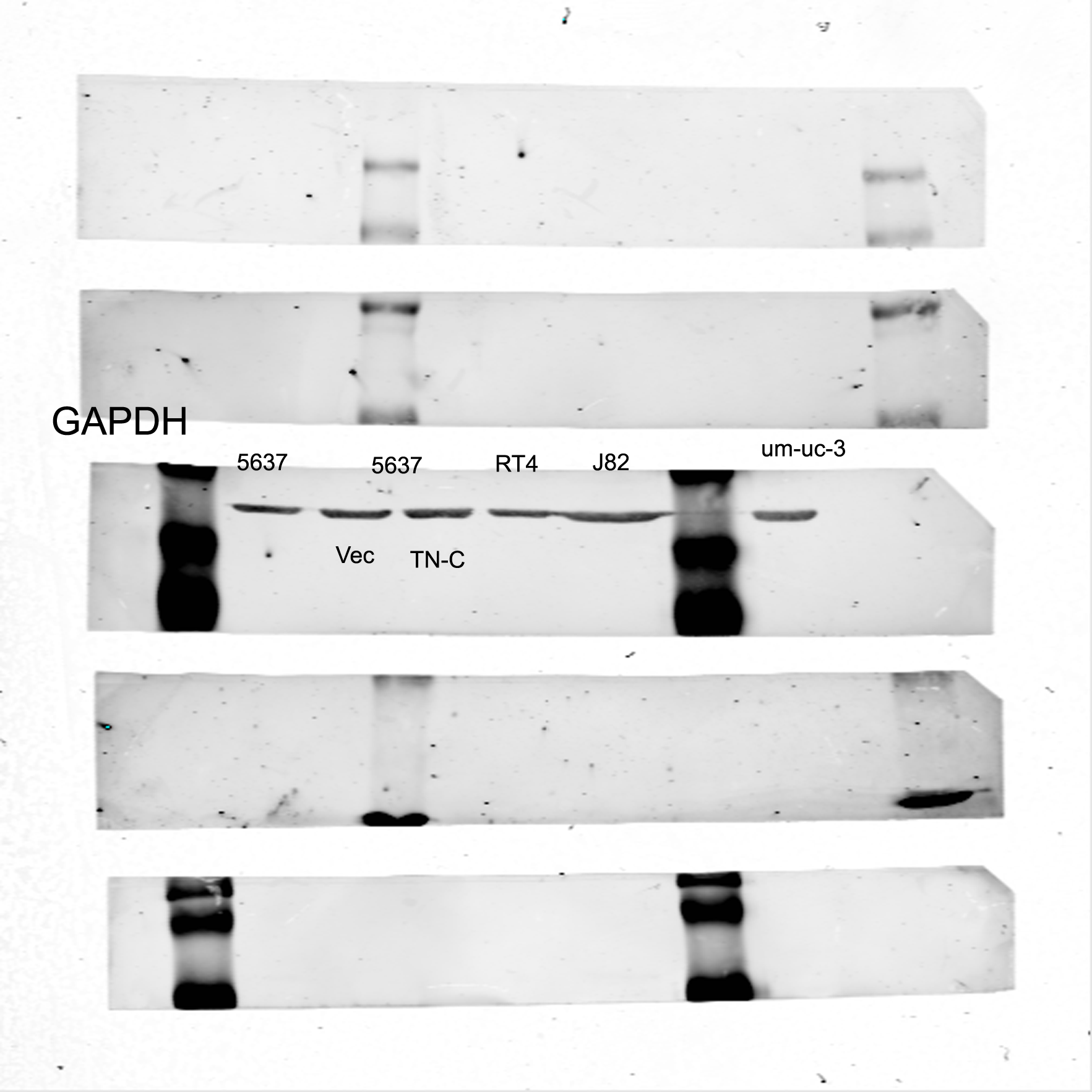

Supplement: Supplementary file 1 — Additional file 1. [file 12885_2022_9285_MOESM1_ESM.zip › Fig2A-5637-GAPDHR3.tif]

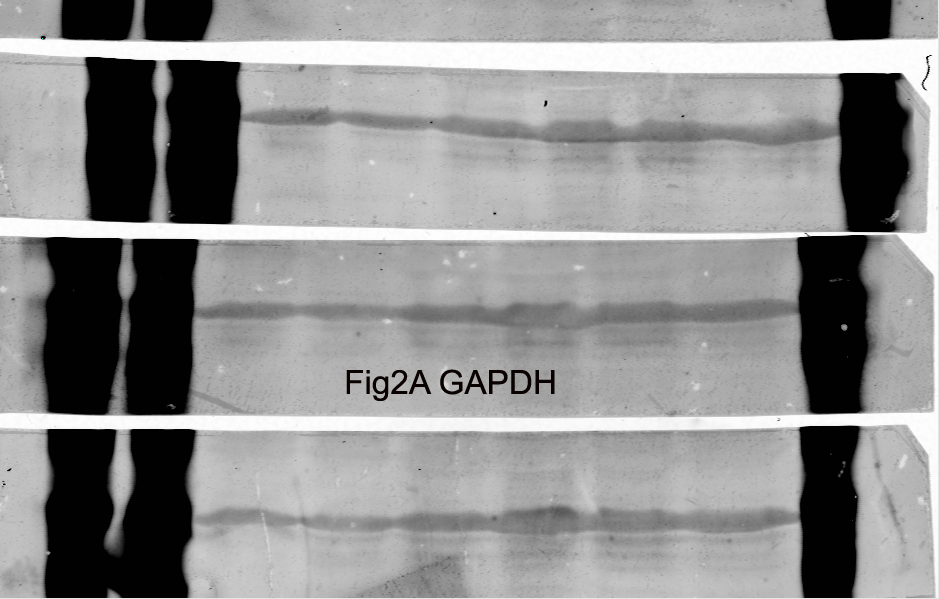

Supplement: Supplementary file 1 — Additional file 1. [file 12885_2022_9285_MOESM1_ESM.zip › Fig2A-GAPDHR3.tif]

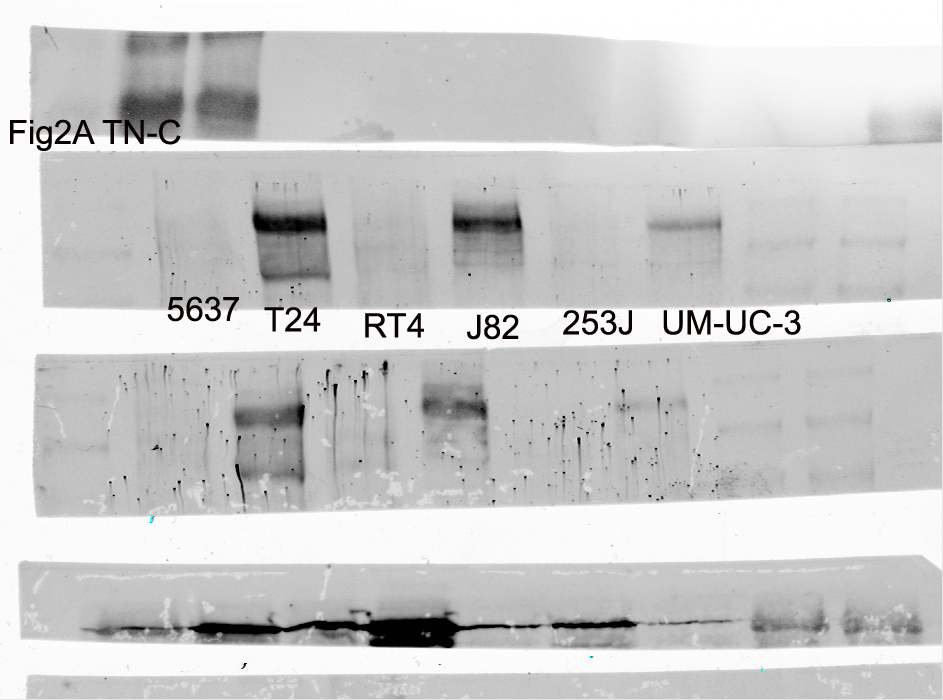

Supplement: Supplementary file 1 — Additional file 1. [file 12885_2022_9285_MOESM1_ESM.zip › Fig2A-TN-CR3.tif]

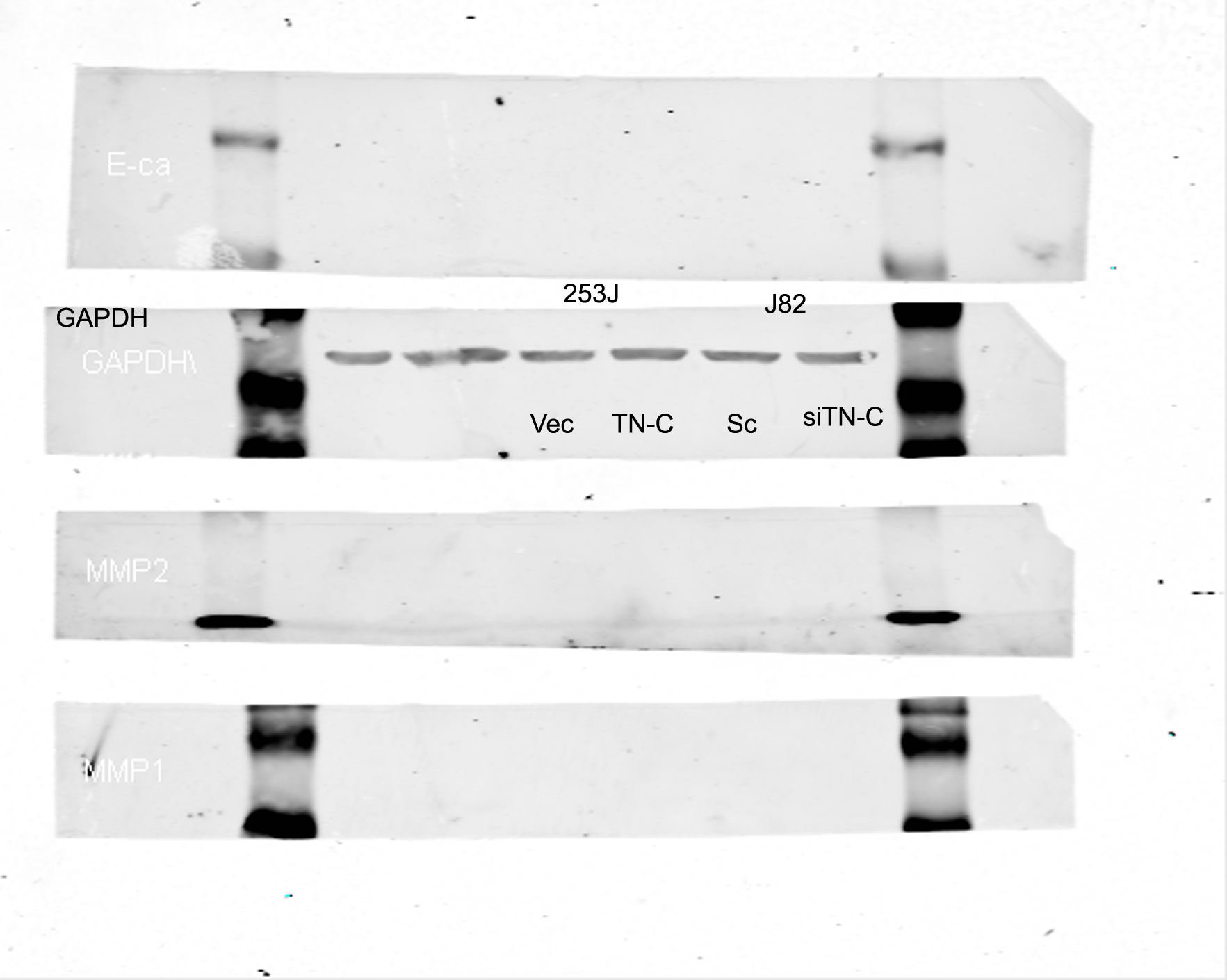

Supplement: Supplementary file 1 — Additional file 1. [file 12885_2022_9285_MOESM1_ESM.zip › Fig2D-253J-GAPDHR3.tif]

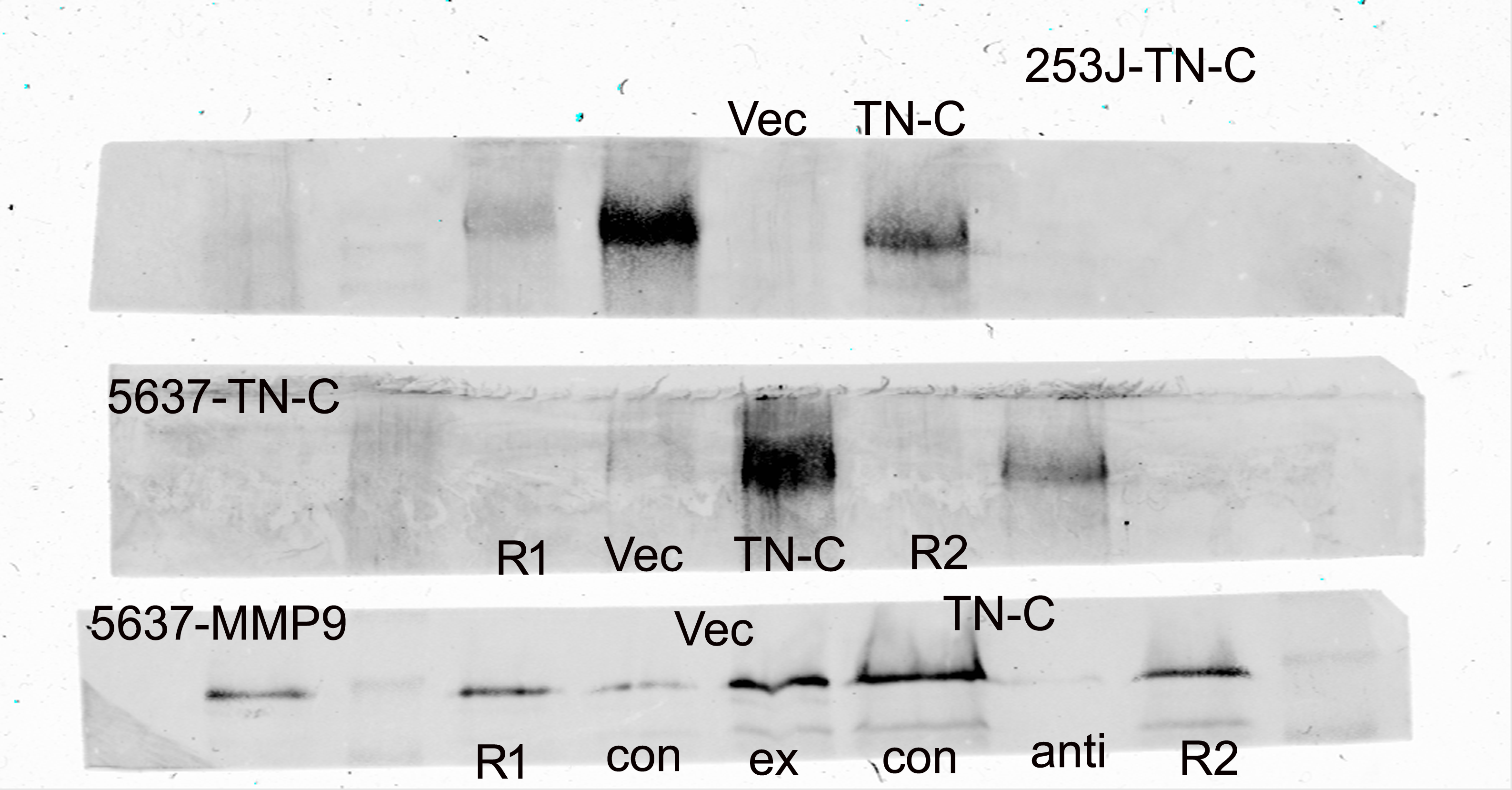

Supplement: Supplementary file 1 — Additional file 1. [file 12885_2022_9285_MOESM1_ESM.zip › Fig2D-253J-TN-CR3.tif]

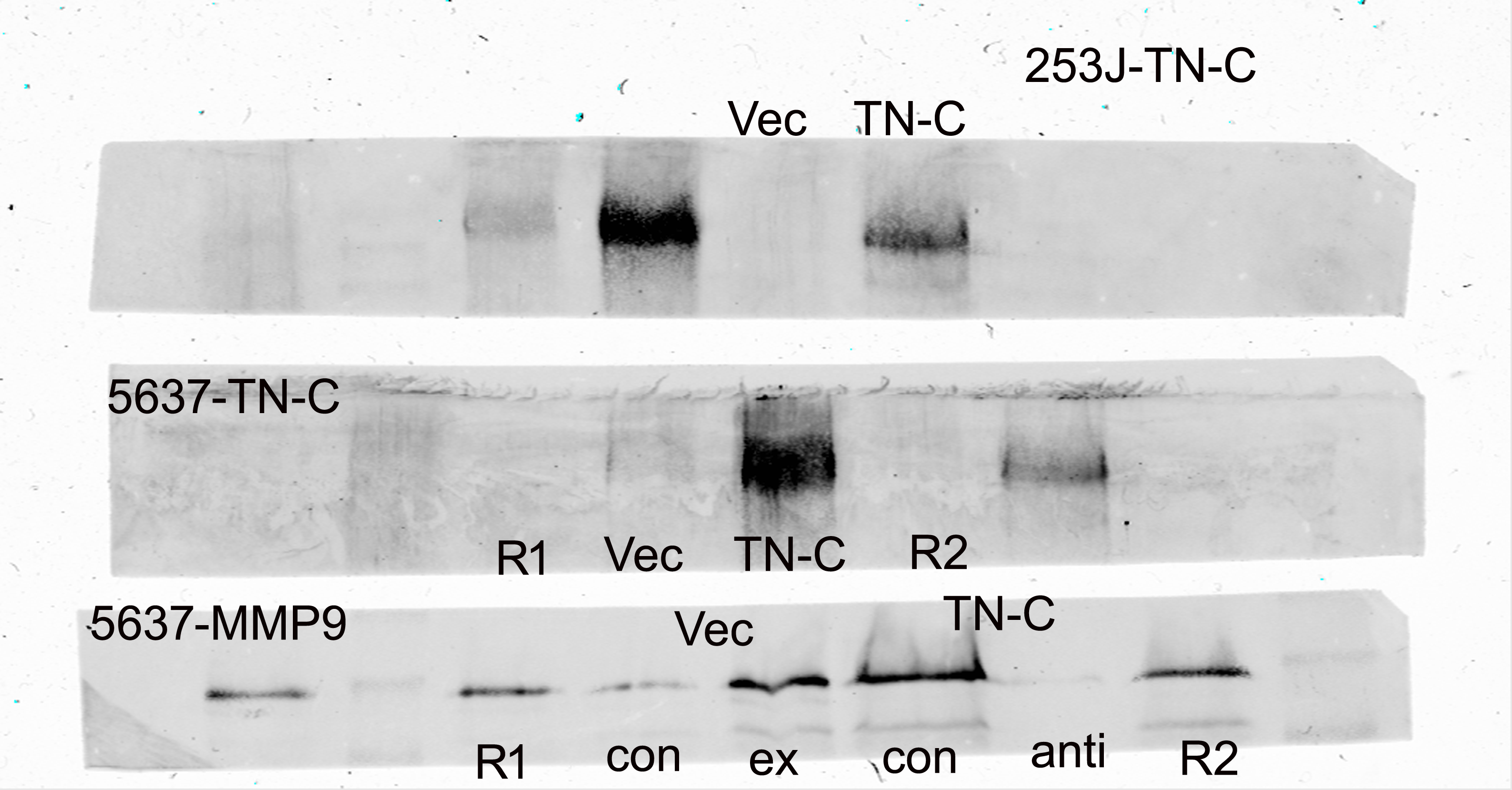

Supplement: Supplementary file 1 — Additional file 1. [file 12885_2022_9285_MOESM1_ESM.zip › Fig2D-5637-TN-CR3.tif]

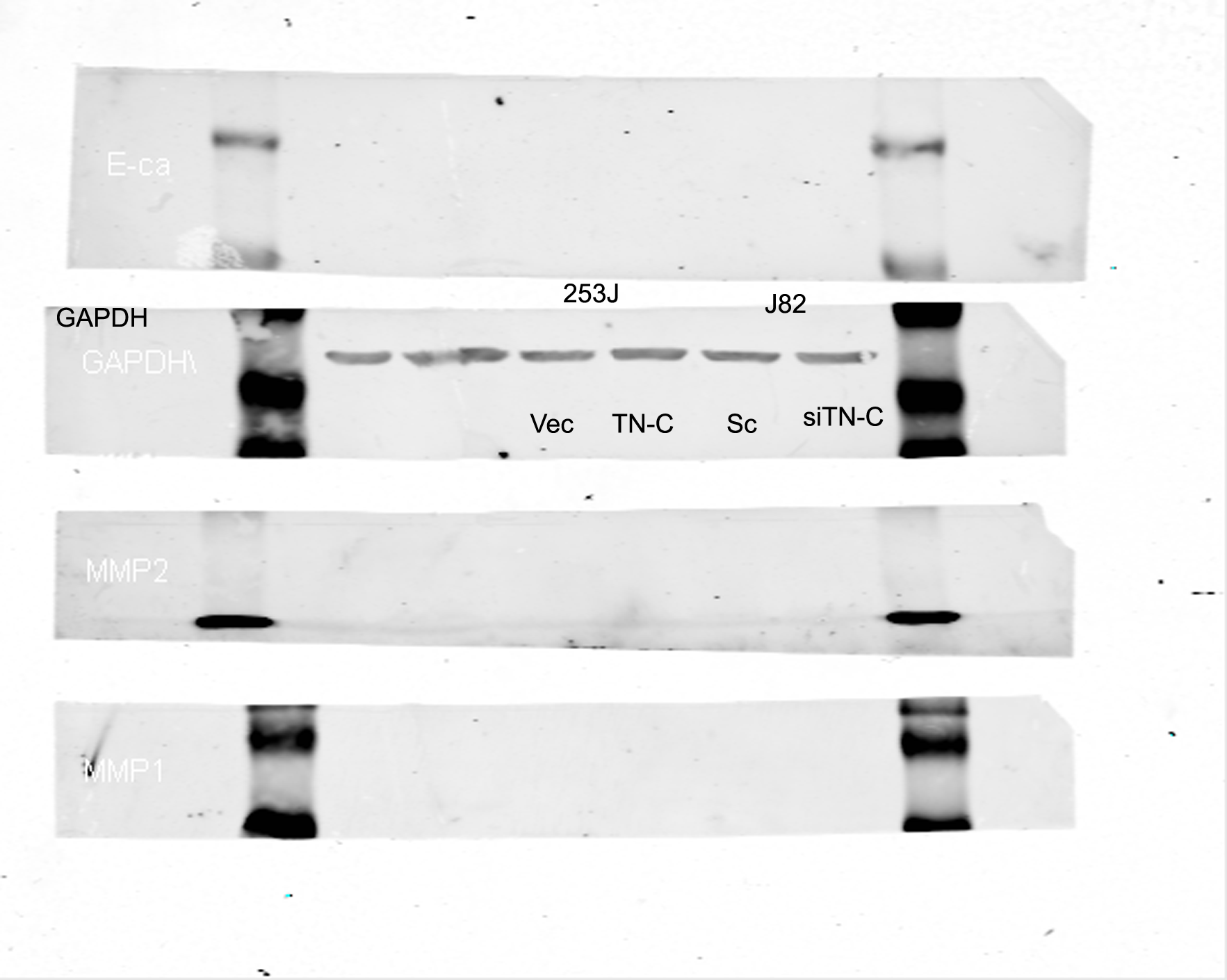

Supplement: Supplementary file 1 — Additional file 1. [file 12885_2022_9285_MOESM1_ESM.zip › Fig2D-J82-GAPDHR3.tif]

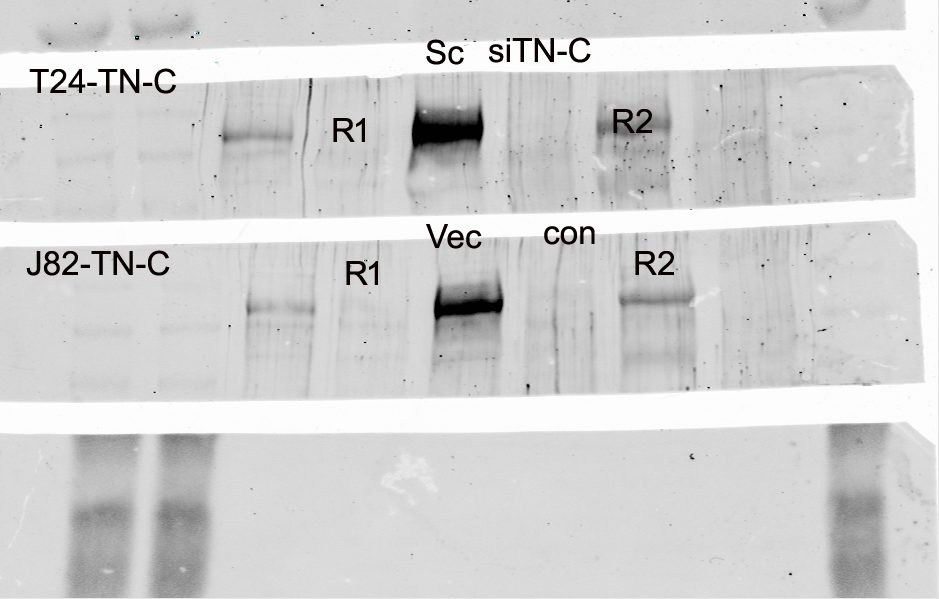

Supplement: Supplementary file 1 — Additional file 1. [file 12885_2022_9285_MOESM1_ESM.zip › Fig2D-J82-TN-CR3.tif]

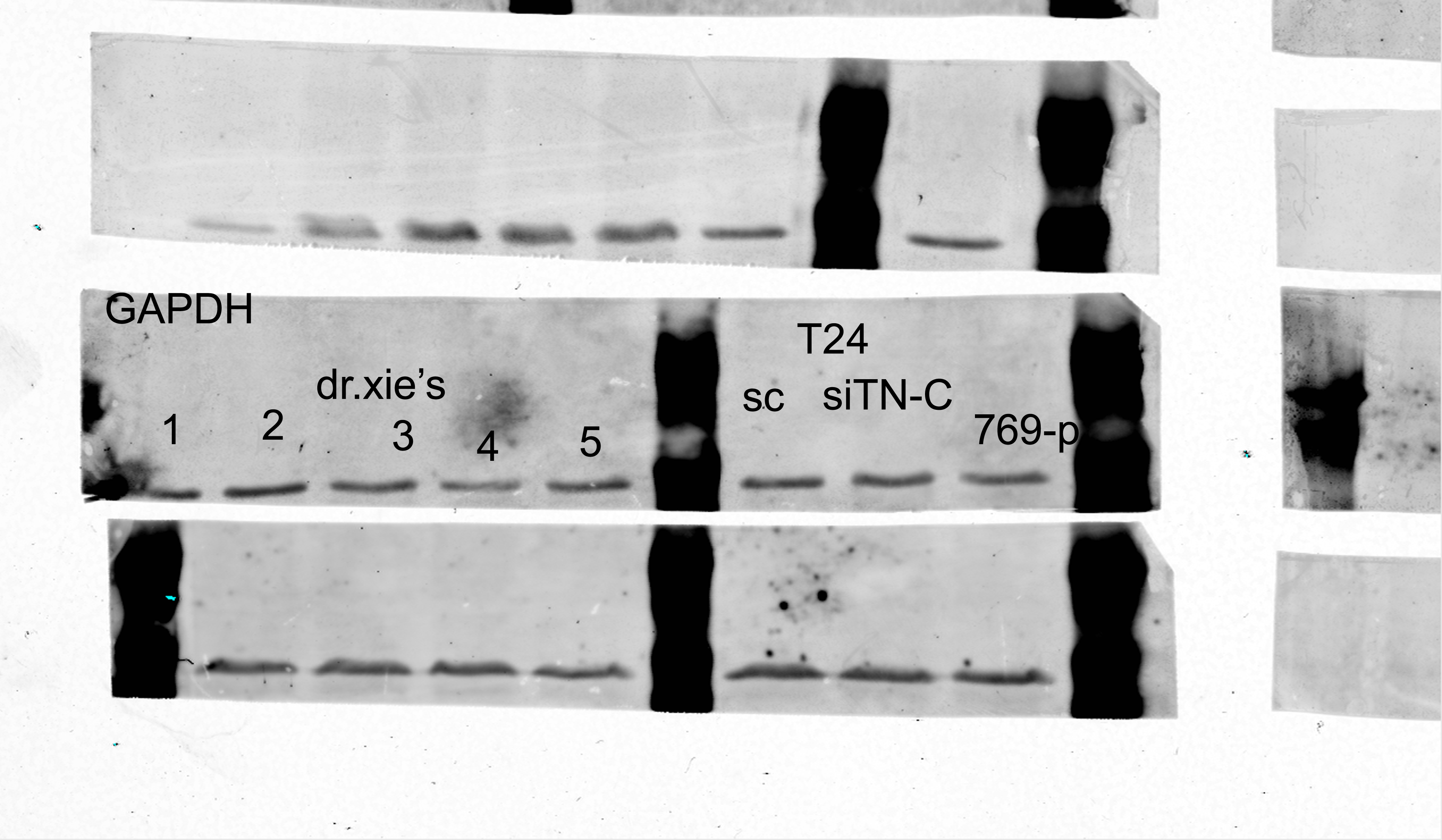

Supplement: Supplementary file 1 — Additional file 1. [file 12885_2022_9285_MOESM1_ESM.zip › Fig2D-T24-GAPDHR3.tif]

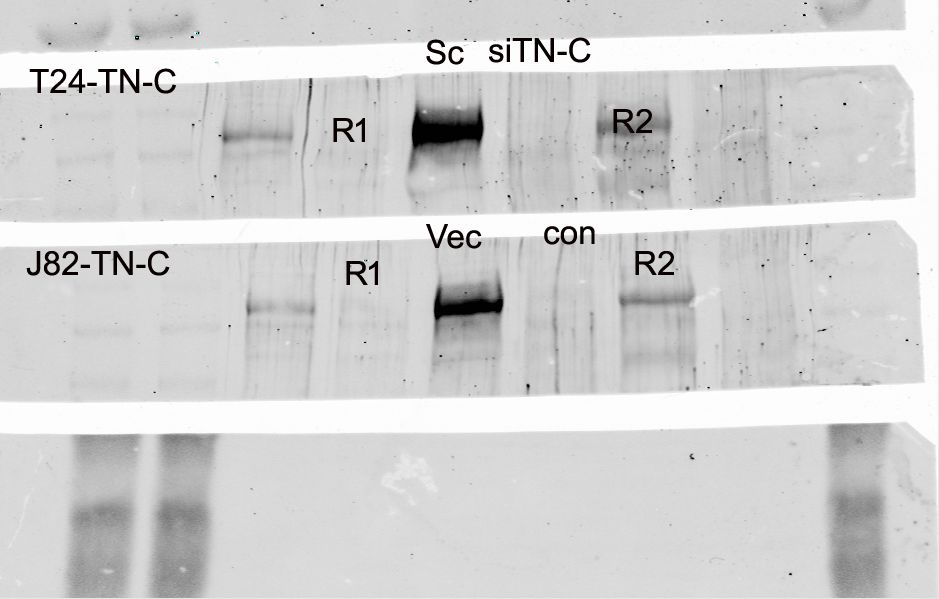

Supplement: Supplementary file 1 — Additional file 1. [file 12885_2022_9285_MOESM1_ESM.zip › Fig2D-T24-TN-CR3.tif]

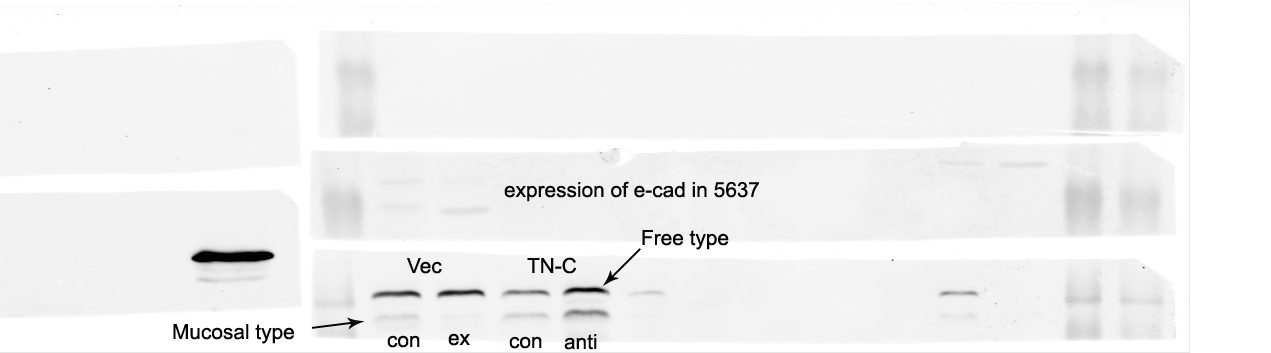

Supplement: Supplementary file 1 — Additional file 1. [file 12885_2022_9285_MOESM1_ESM.zip › Fig5A-5637-E-CadR3.tif]

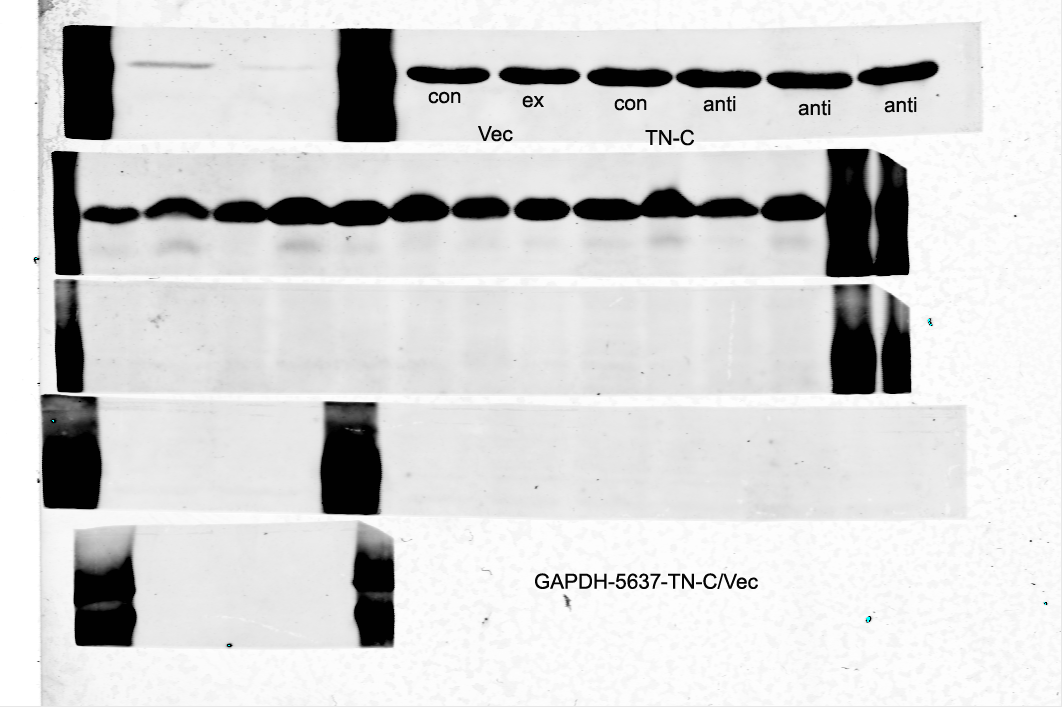

Supplement: Supplementary file 1 — Additional file 1. [file 12885_2022_9285_MOESM1_ESM.zip › Fig5A-5637-GAPDHR3.tif]

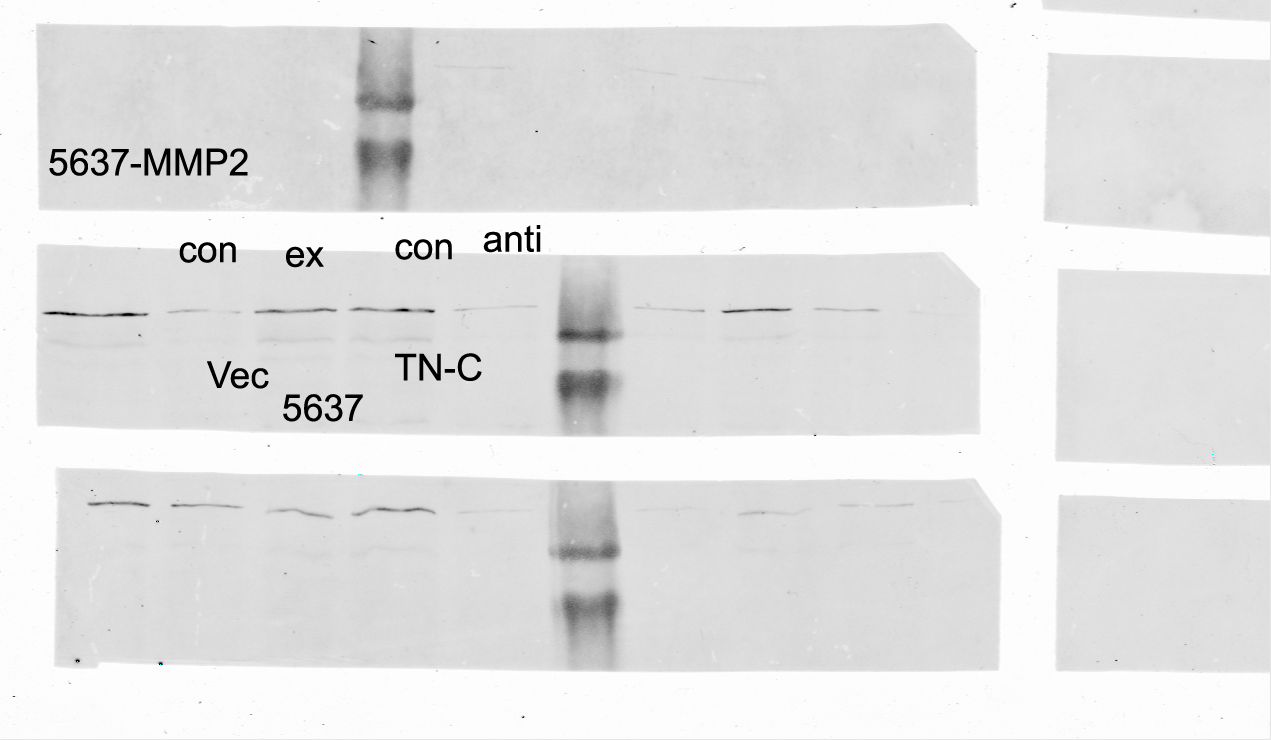

Supplement: Supplementary file 1 — Additional file 1. [file 12885_2022_9285_MOESM1_ESM.zip › Fig5A-5637-MMP2R3.tif]

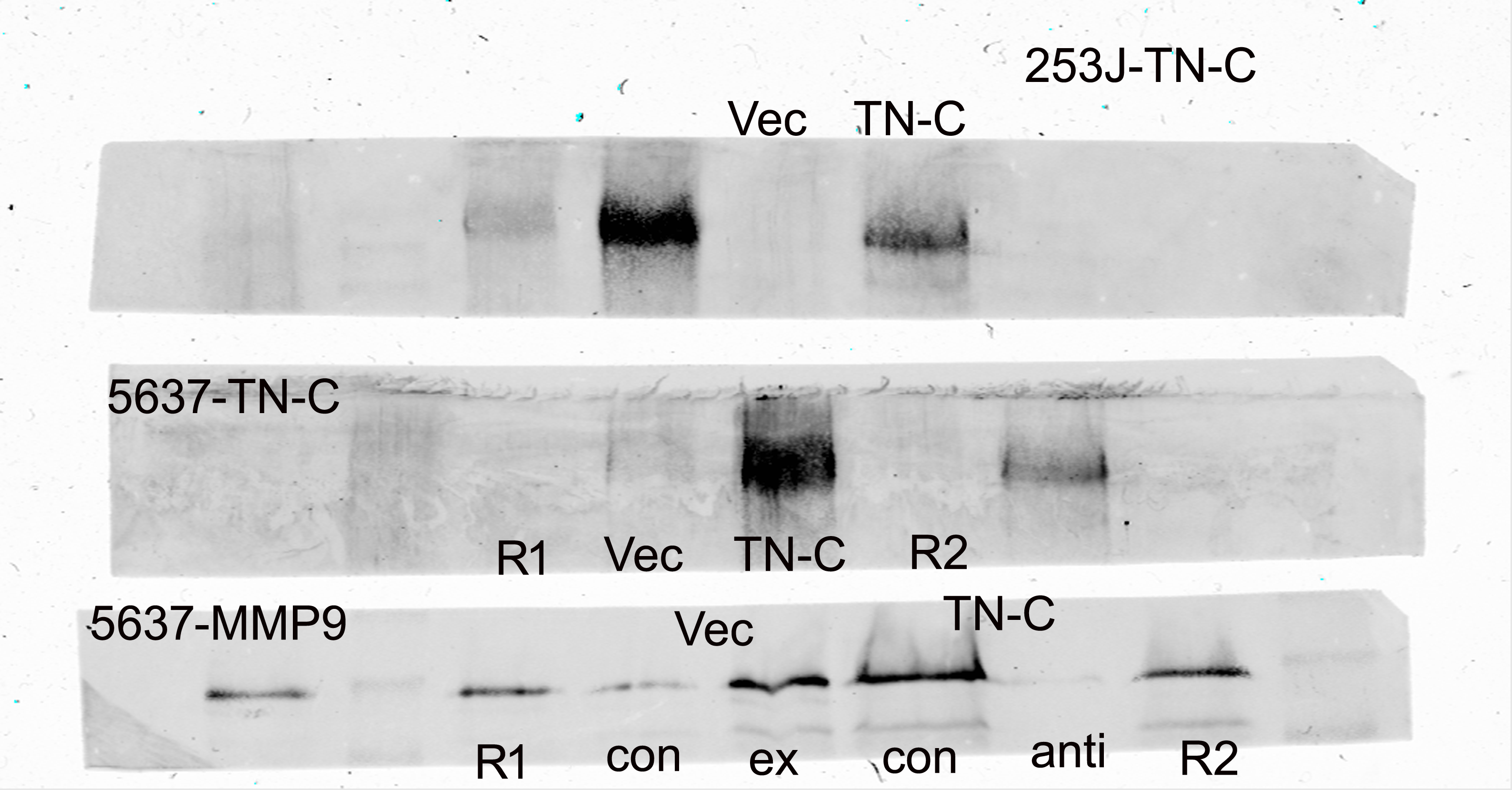

Supplement: Supplementary file 1 — Additional file 1. [file 12885_2022_9285_MOESM1_ESM.zip › Fig5A-5637-MMP9R3.tif]

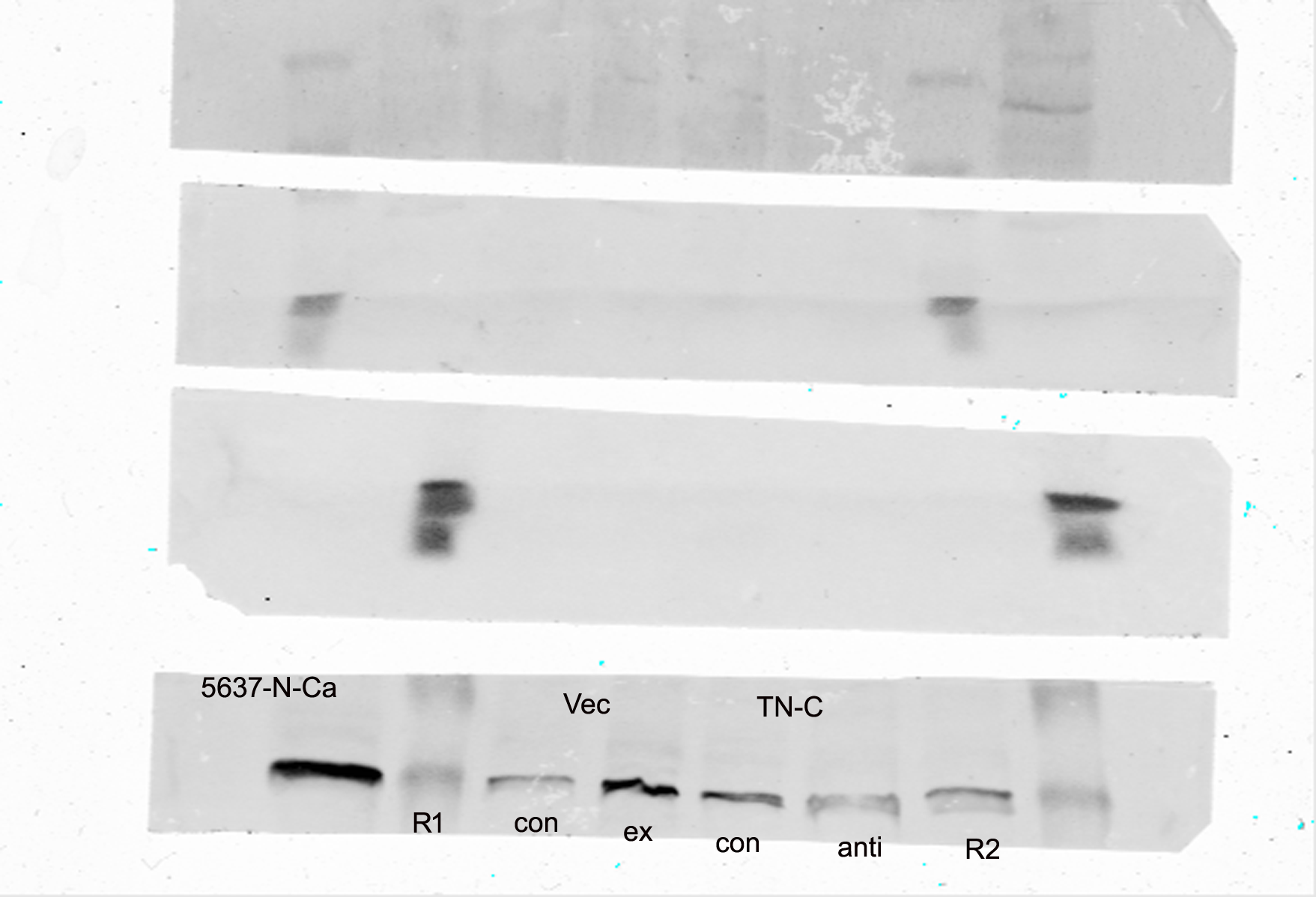

Supplement: Supplementary file 1 — Additional file 1. [file 12885_2022_9285_MOESM1_ESM.zip › Fig5A-5637-N-CaR3.tif]

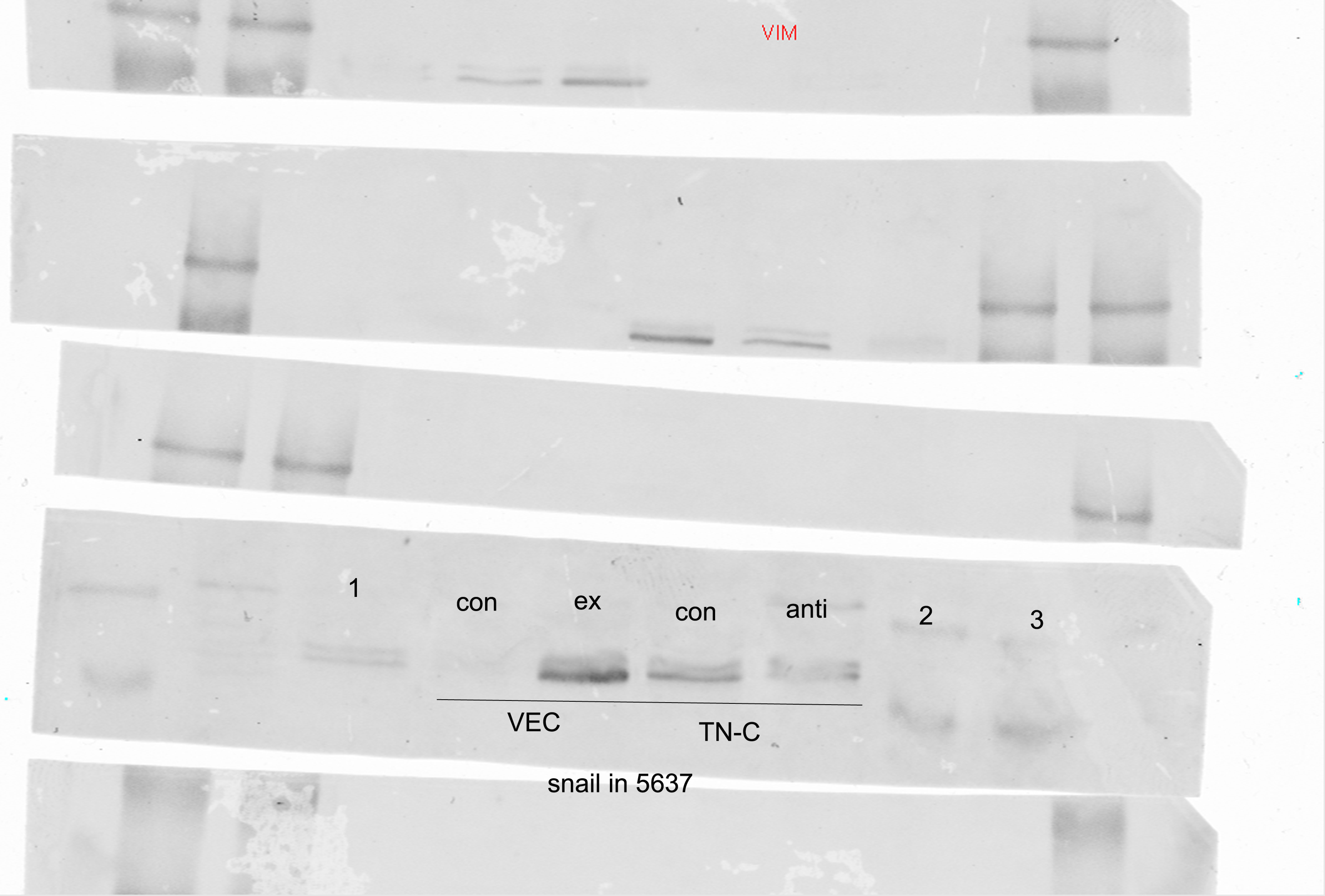

Supplement: Supplementary file 1 — Additional file 1. [file 12885_2022_9285_MOESM1_ESM.zip › Fig5A-5637-snailR3.tif]

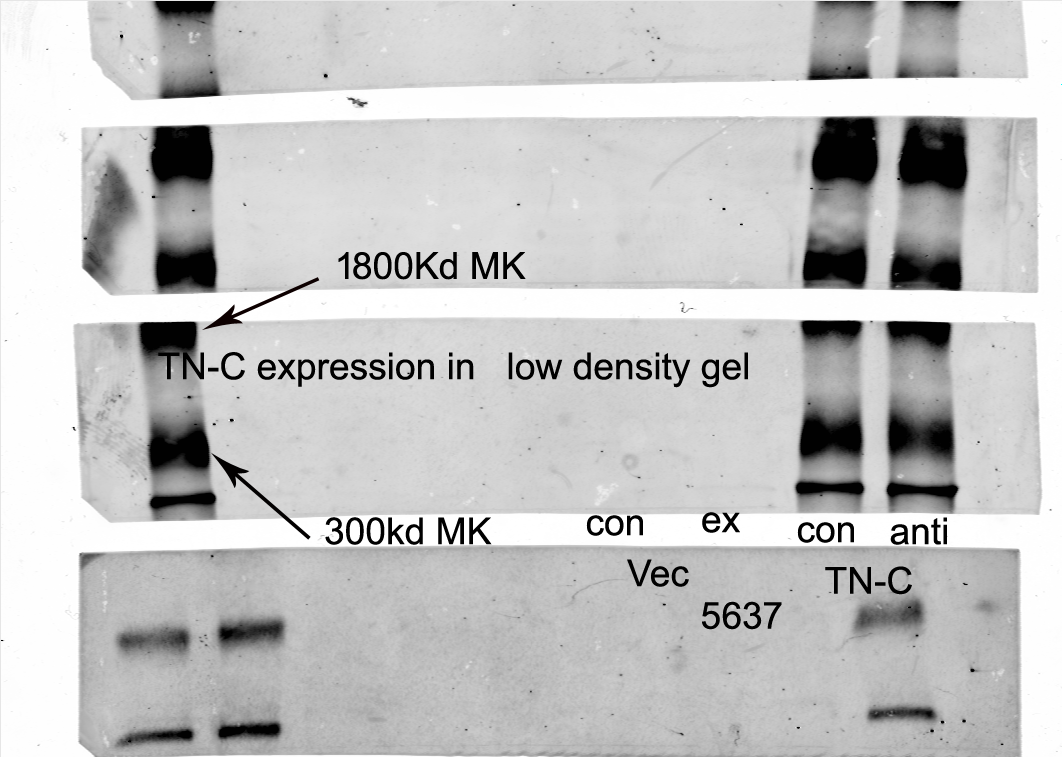

Supplement: Supplementary file 1 — Additional file 1. [file 12885_2022_9285_MOESM1_ESM.zip › Fig5A-5637-TN-CR3.tif]

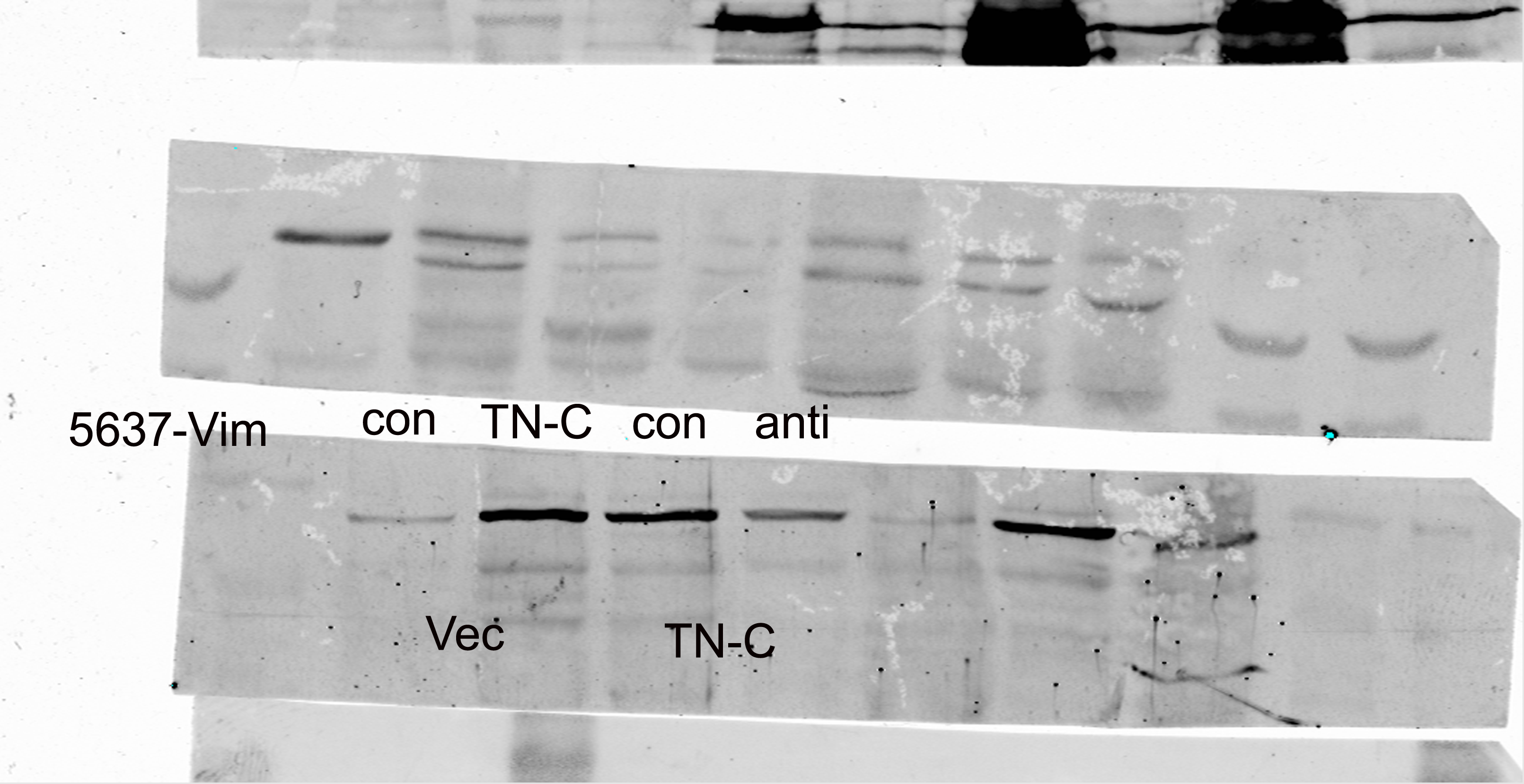

Supplement: Supplementary file 1 — Additional file 1. [file 12885_2022_9285_MOESM1_ESM.zip › Fig5A-5637-vimR3.tif]

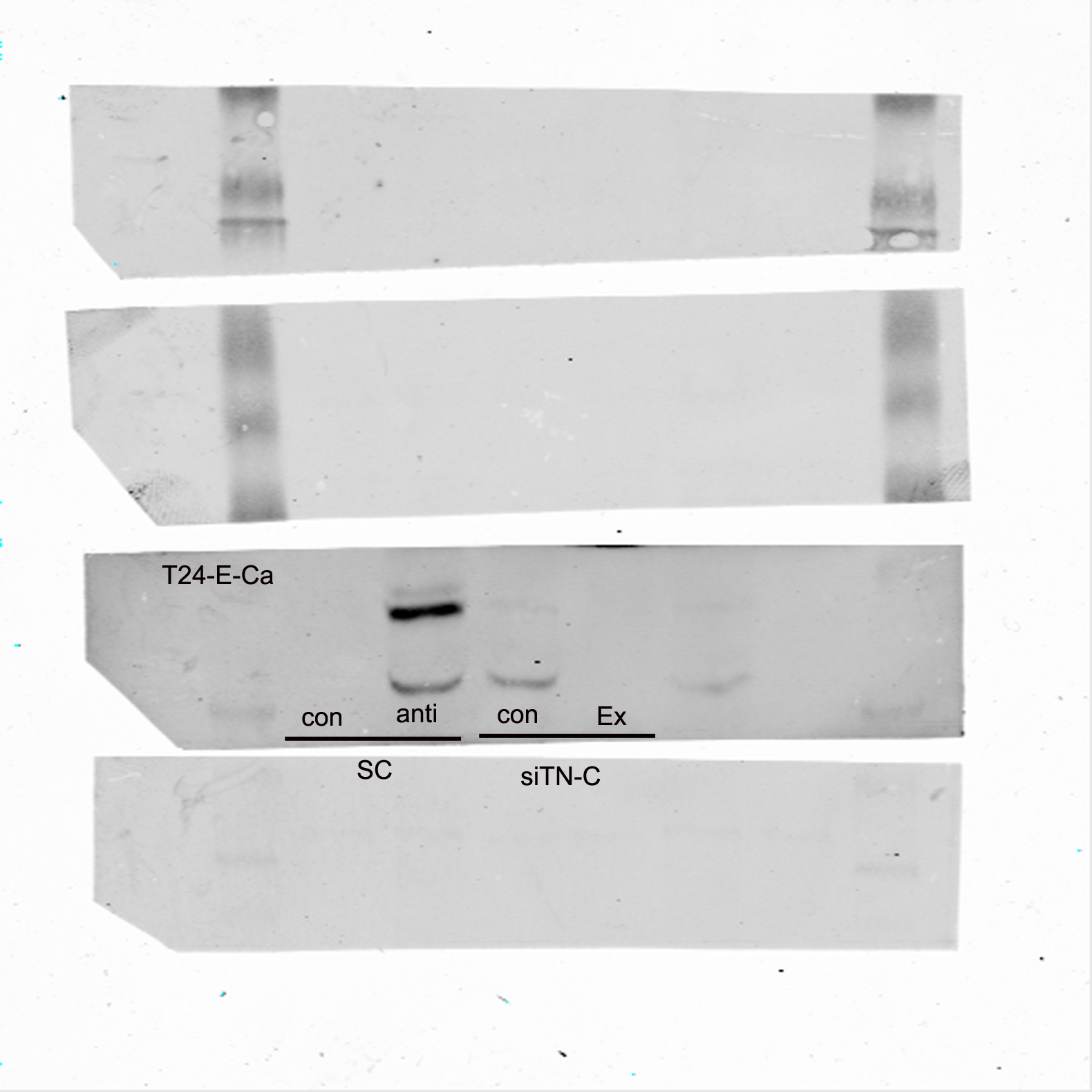

Supplement: Supplementary file 1 — Additional file 1. [file 12885_2022_9285_MOESM1_ESM.zip › Fig5A-T24-E-CadR3.tif]

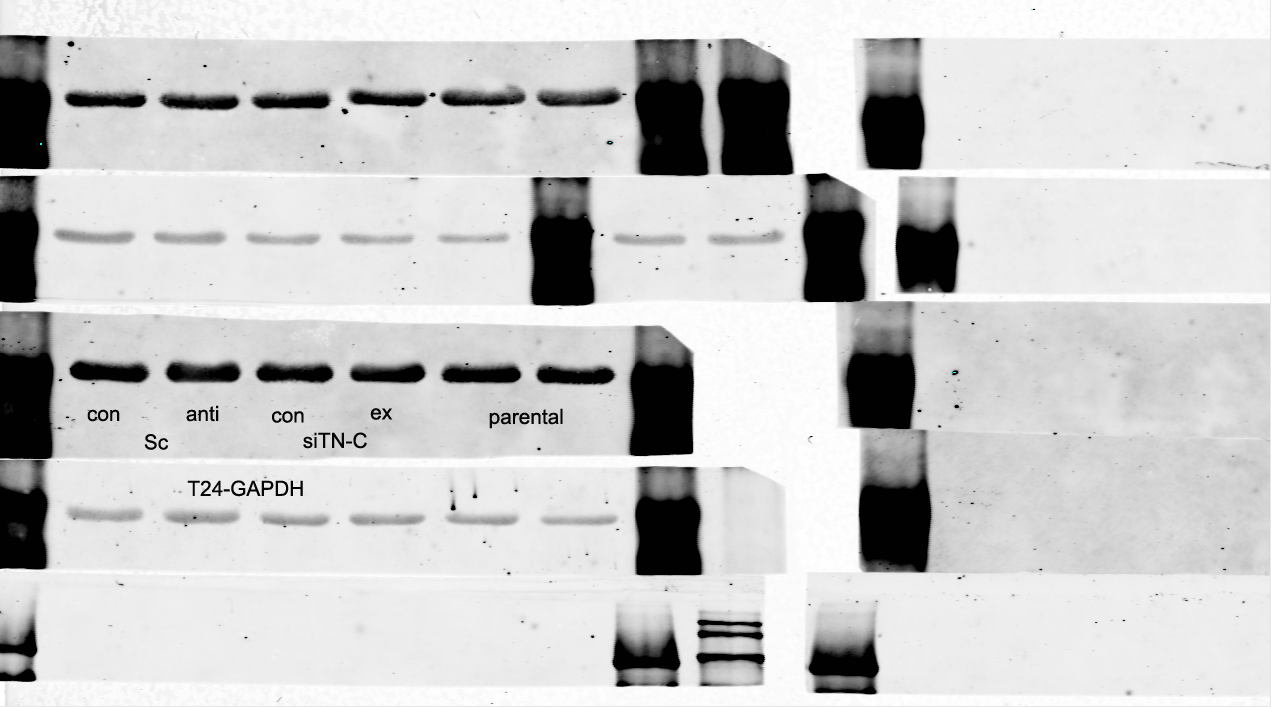

Supplement: Supplementary file 1 — Additional file 1. [file 12885_2022_9285_MOESM1_ESM.zip › Fig5A-T24-GAPDHR3.tif]

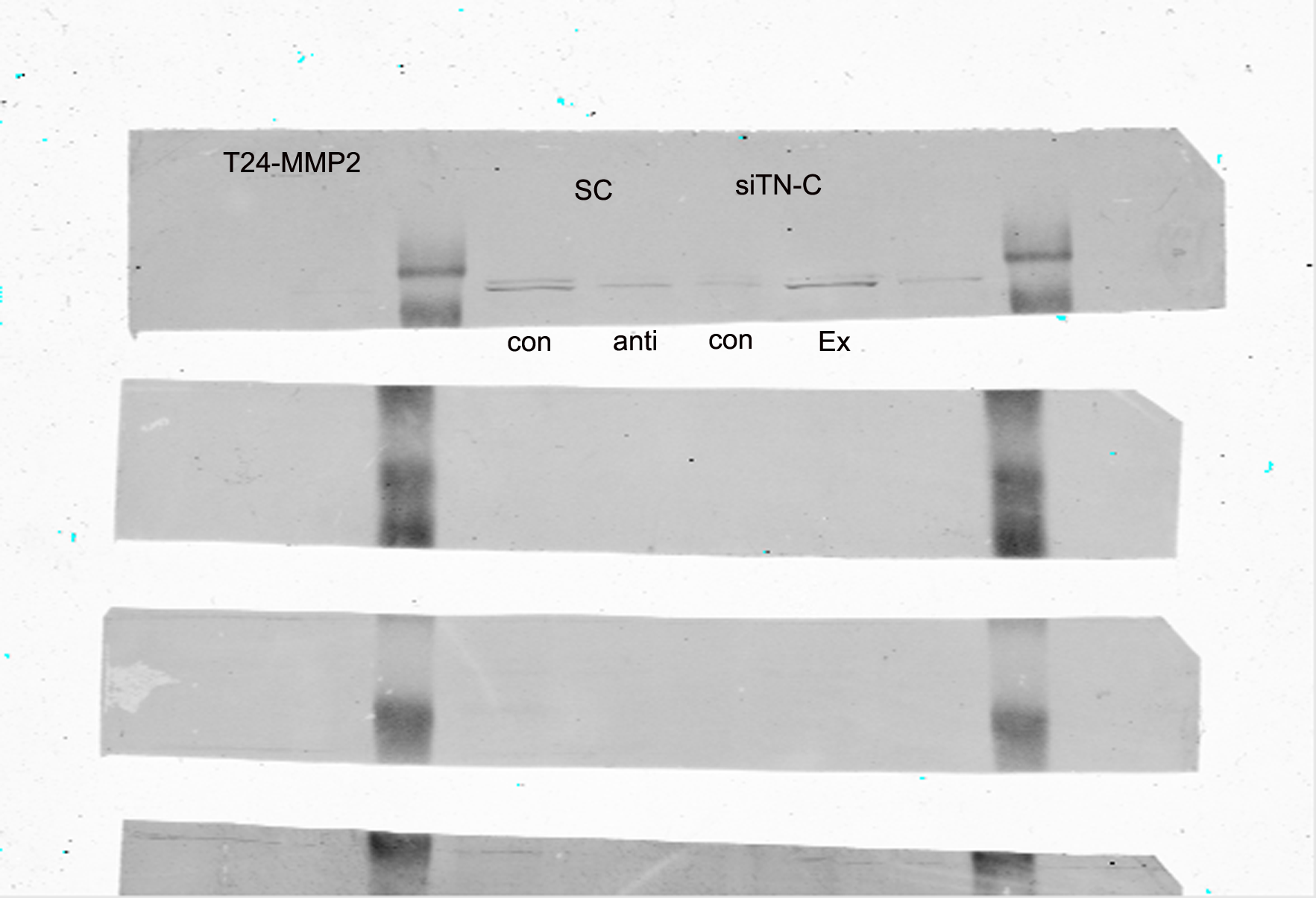

Supplement: Supplementary file 1 — Additional file 1. [file 12885_2022_9285_MOESM1_ESM.zip › Fig5A-T24-MMP2R3.tif]

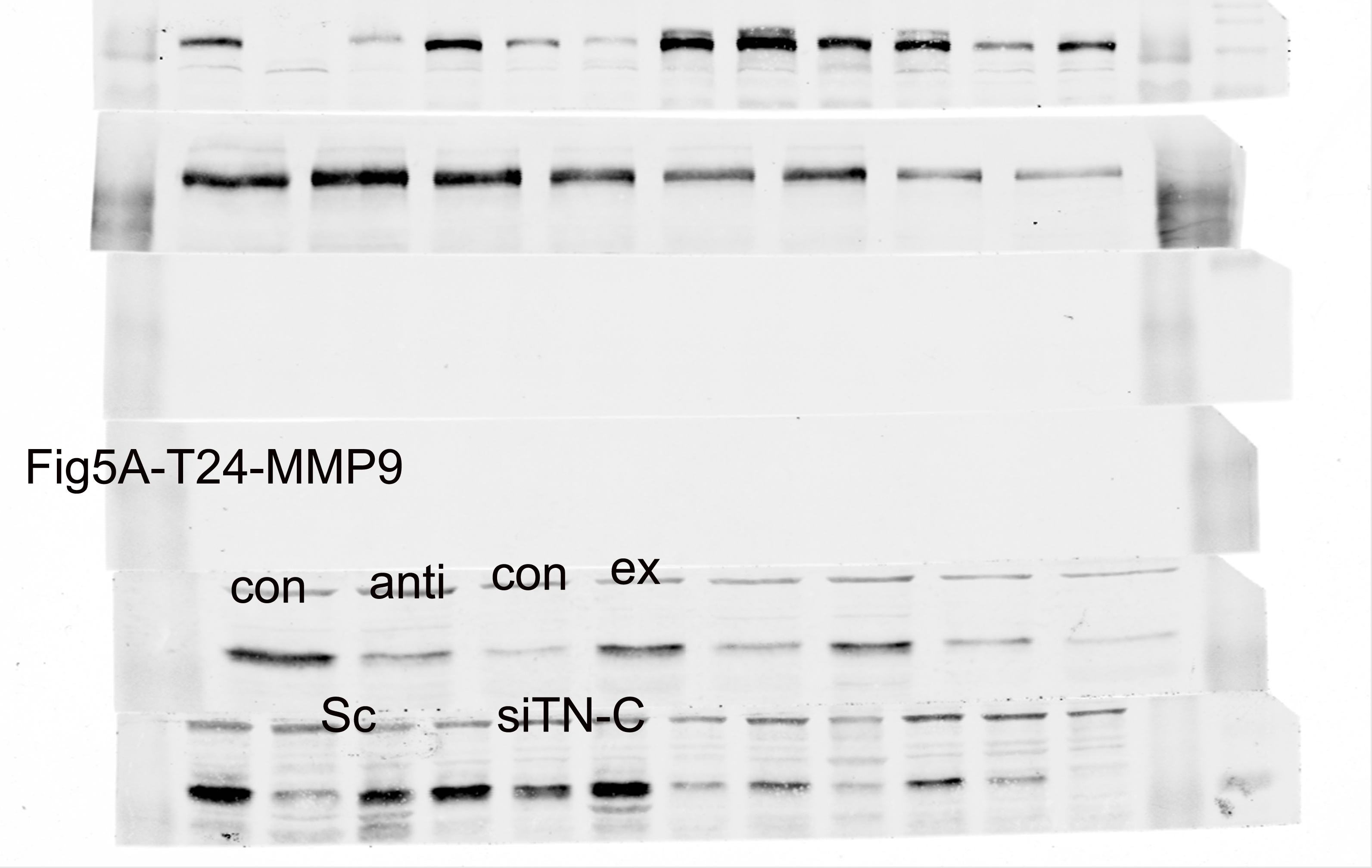

Supplement: Supplementary file 1 — Additional file 1. [file 12885_2022_9285_MOESM1_ESM.zip › Fig5A-T24-MMP9R3.tif]

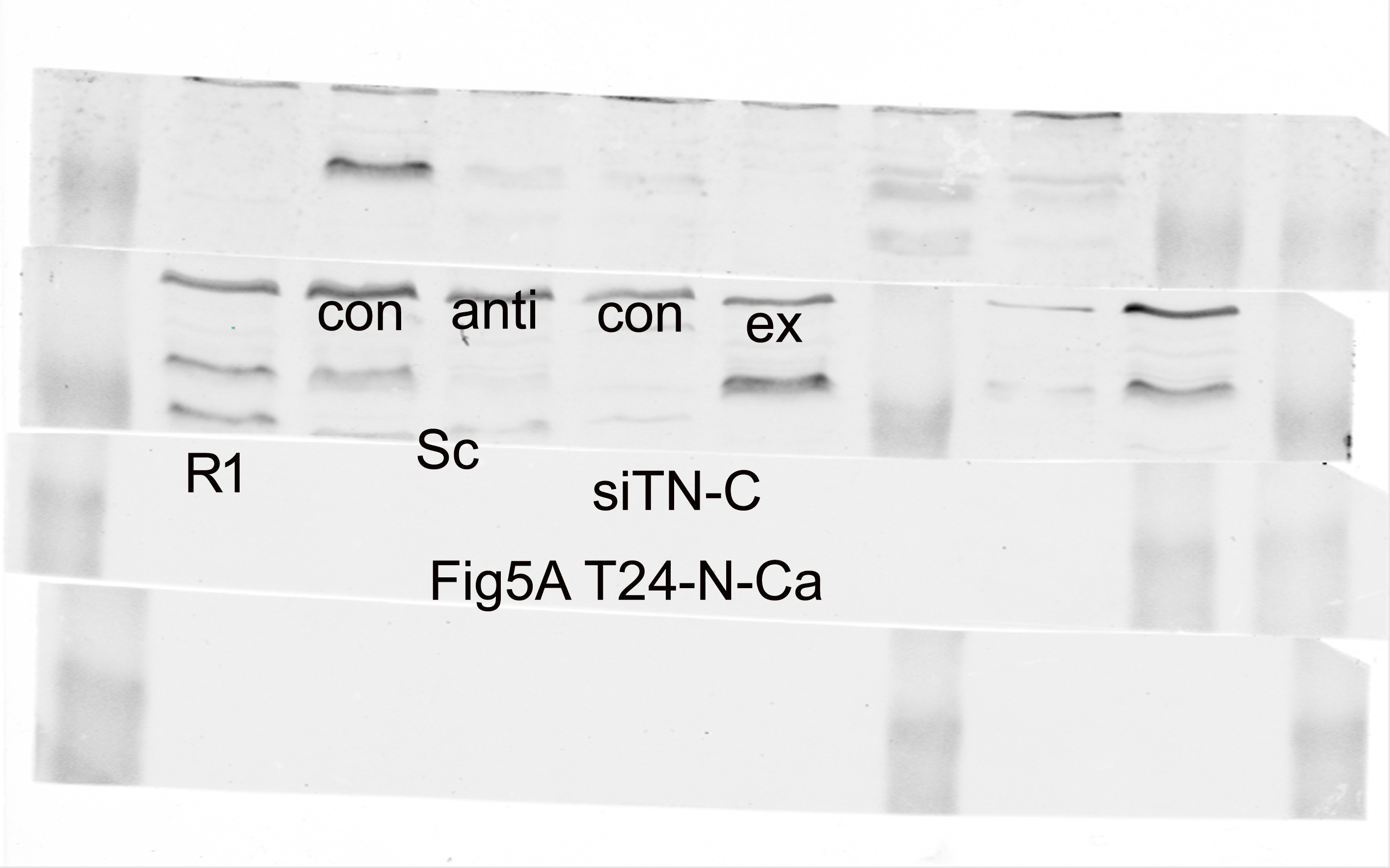

Supplement: Supplementary file 1 — Additional file 1. [file 12885_2022_9285_MOESM1_ESM.zip › Fig5A-T24-N-CadR3.tif]

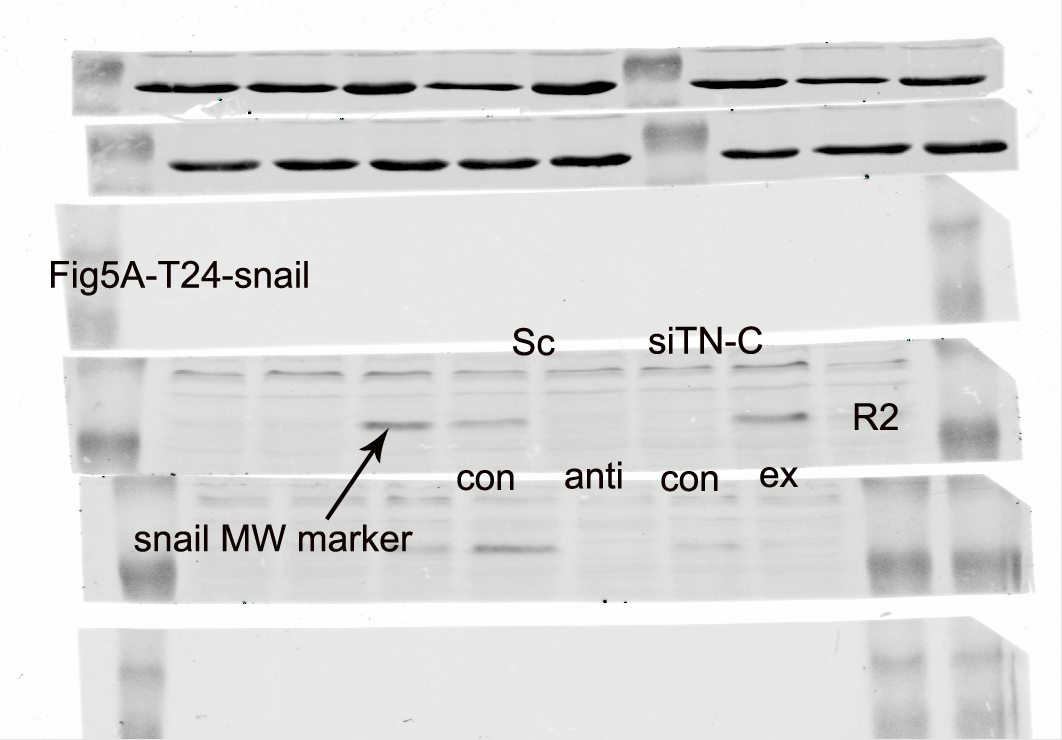

Supplement: Supplementary file 1 — Additional file 1. [file 12885_2022_9285_MOESM1_ESM.zip › Fig5A-T24-snailR3.tif]

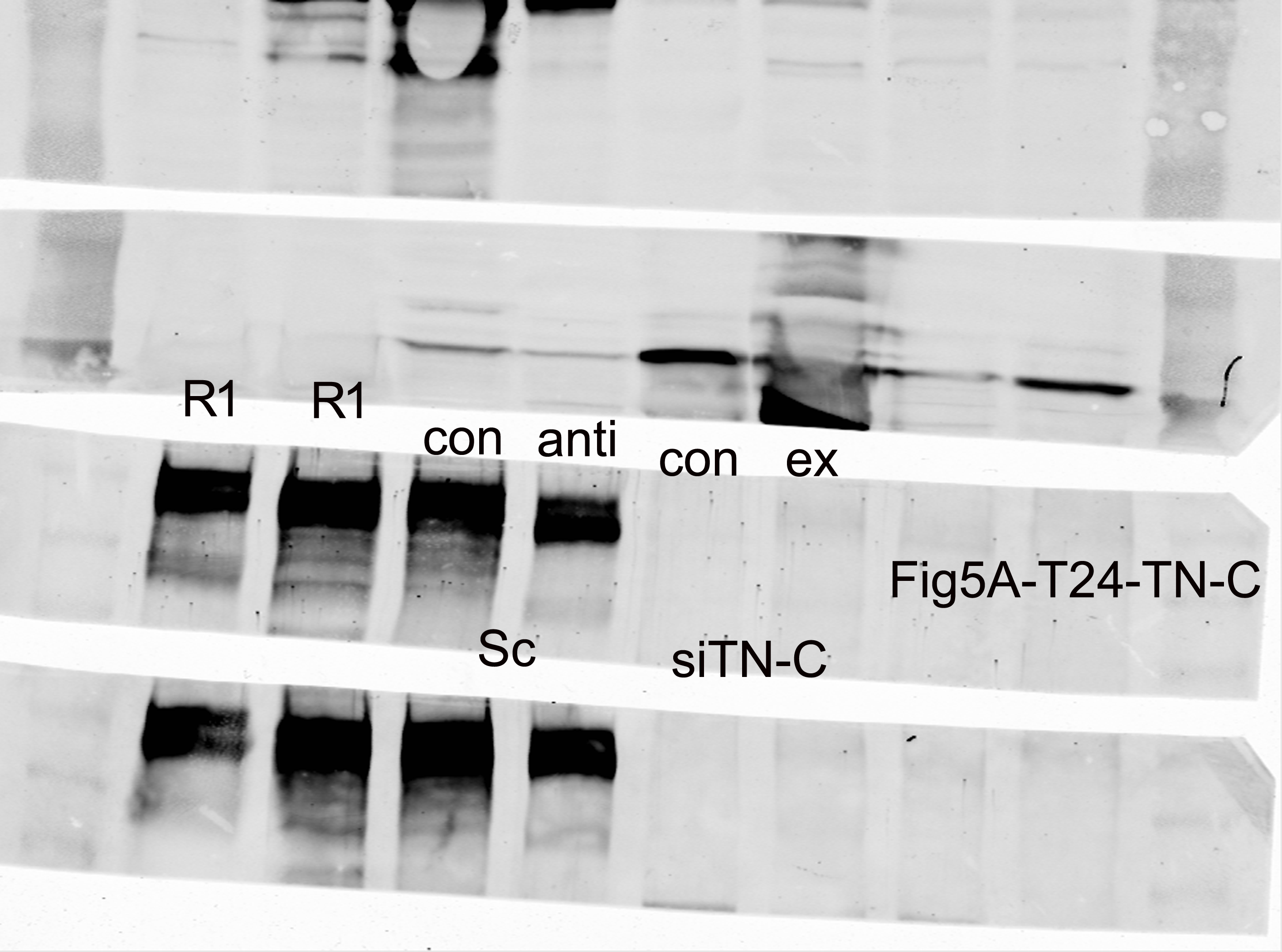

Supplement: Supplementary file 1 — Additional file 1. [file 12885_2022_9285_MOESM1_ESM.zip › Fig5A-T24-TN-CR3.tif]

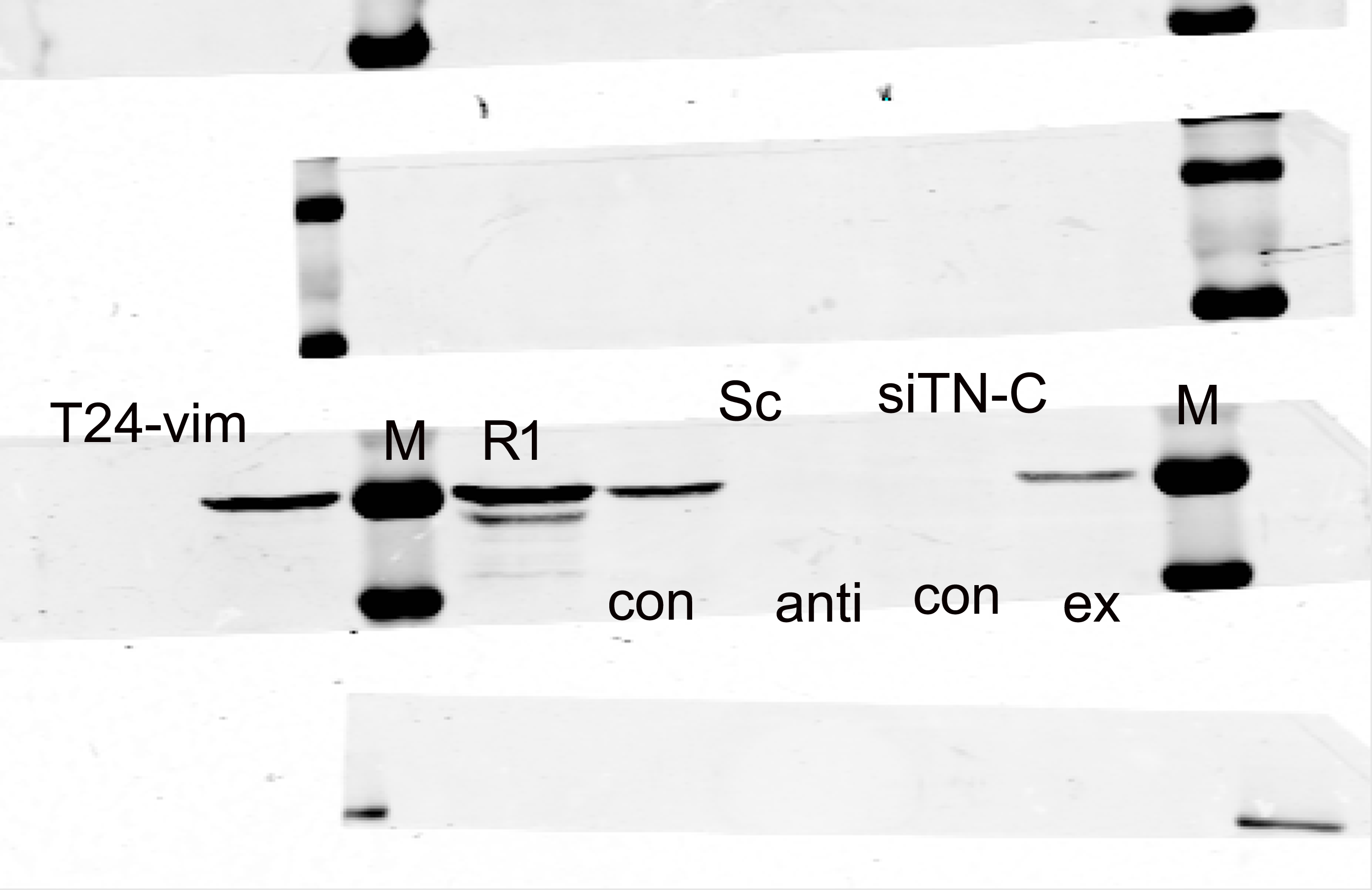

Supplement: Supplementary file 1 — Additional file 1. [file 12885_2022_9285_MOESM1_ESM.zip › Fig5A-T24-vimR3.tif]

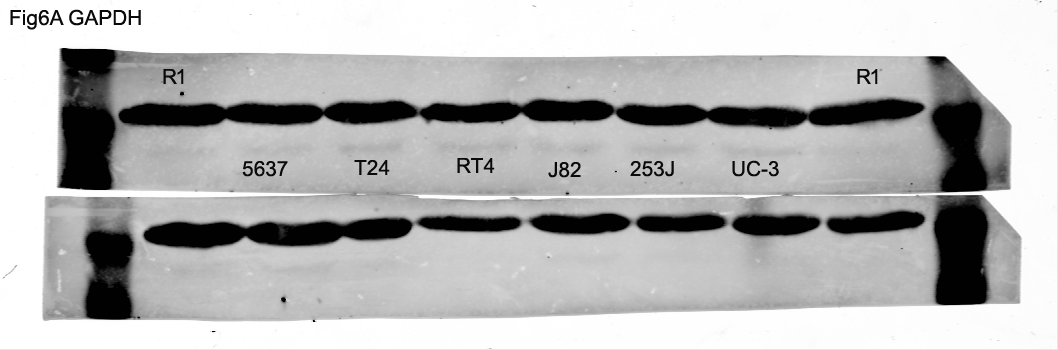

Supplement: Supplementary file 1 — Additional file 1. [file 12885_2022_9285_MOESM1_ESM.zip › Fig6A-GAPDHR3.tif]

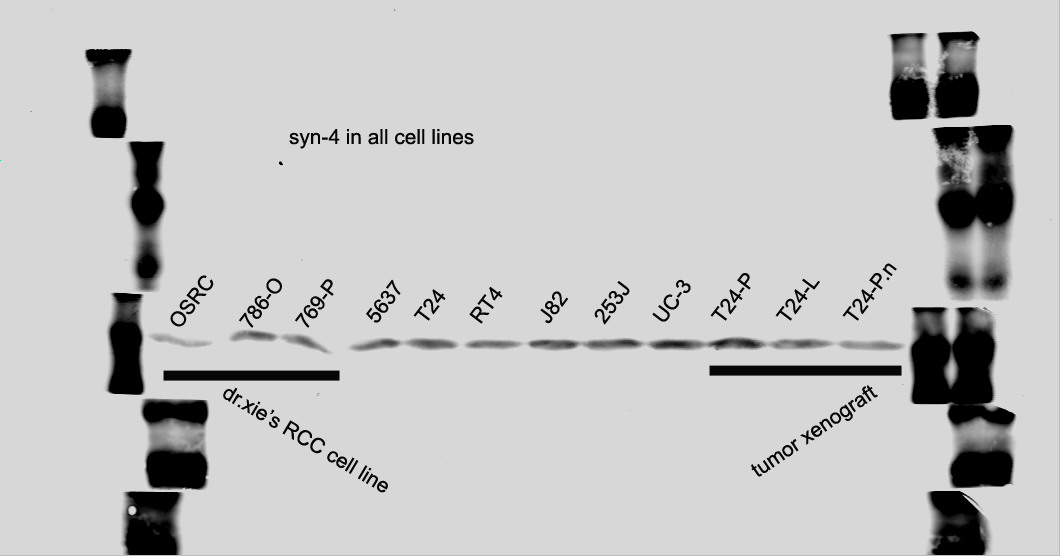

Supplement: Supplementary file 1 — Additional file 1. [file 12885_2022_9285_MOESM1_ESM.zip › Fig6A-syn-4R3.tif]

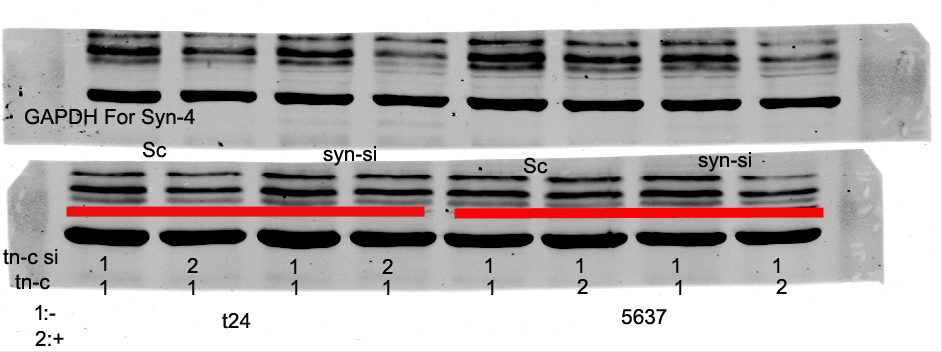

Supplement: Supplementary file 1 — Additional file 1. [file 12885_2022_9285_MOESM1_ESM.zip › Fig6B-GAPDHR3.tif]

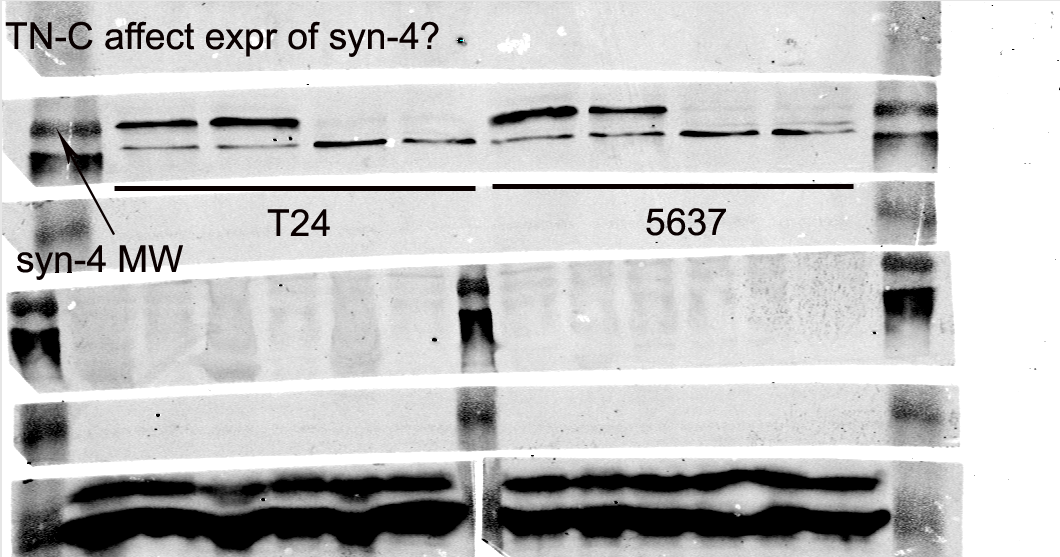

Supplement: Supplementary file 1 — Additional file 1. [file 12885_2022_9285_MOESM1_ESM.zip › Fig6B-syn-4R3.tif]
